# Evaluation of Neurologic and Psychiatric Outcomes After Hospital Discharge Among Adult Survivors of Cardiac Arrest

**DOI:** 10.1001/jamanetworkopen.2022.13546

**Published:** 2022-05-31

**Authors:** Niels Secher, Kasper Adelborg, Péter Szentkúti, Christian Fynbo Christiansen, Asger Granfeldt, Victor W. Henderson, Henrik Toft Sørensen

**Affiliations:** 1 Department of Clinical Epidemiology, Aarhus University Hospital and Aarhus University, Aarhus, Denmark; 2 Department of Anesthesiology and Intensive Care Medicine, Horsens Regional Hospital, Horsens, Denmark; 3 Department of Anesthesiology and Intensive Care Medicine, Aarhus University Hospital, Aarhus, Denmark; 4 Department of Clinical Biochemistry, Aarhus University Hospital, Aarhus, Denmark; 5 Department of Epidemiology and Population Health, Stanford University, Stanford, California; 6 Department of Neurology and Neurological Sciences, Stanford University, Stanford, California; 7 Clinical Excellence Research Center, Stanford University, Stanford, California

## Abstract

**Importance:**

Long-term risks of neurologic and psychiatric disease after cardiac arrest are largely unknown.

**Objective:**

To examine the short-term and long-term risks of common neurologic outcomes (stroke, epilepsy, Parkinson disease, and dementia) and psychiatric outcomes (depression and anxiety) in patients after hospitalization for cardiac arrest.

**Design, Setting, and Participants:**

This nationwide population-based cohort study with 21 years of follow-up included data on 250 838 adults from all Danish hospitals between January 1, 1996, and December 31, 2016. Danish medical registries were used to identify all patients with a first-time diagnosis of cardiac arrest and 2 matched comparison cohorts. The first comparison cohort included patients with a first-time diagnosis of myocardial infarction; the second comprised people from the general population. Data analysis was performed from November 1, 2020, to June 30, 2021.

**Exposures:**

In-hospital or out-of-hospital cardiac arrest.

**Main Outcomes and Measures:**

Neurologic and psychiatric outcomes after hospital discharge were ascertained using medical registries. Twenty-one–year hazard ratios (HRs) and 95% CIs were computed based on Cox regression analysis, controlled for matching factors, and adjusted for comorbidity and socioeconomic status.

**Results:**

Among the 250 838 individuals included in this study (median age, 67 years [IQR, 57-76 years]; 173 946 [69.3%] male), 3 groups were identified: 12 046 patients with cardiac arrest, 118 332 patients with myocardial infarction, and 120 460 people from the general population. Compared with patients with myocardial infarction, patients with cardiac arrest had an increased rate of ischemic stroke (10 per 1000 persons; HR, 1.30; 95% CI, 1.02-1.64) and hemorrhagic stroke (2 per 1000 persons; HR, 2.03; 95% CI, 1.12-3.67) in the first year after discharge. During the full follow-up period, rates were as follows: for epilepsy, 28 per 1000 persons (HR, 2.01; 95% CI, 1.66-2.44); for dementia, 73 per 1000 persons (HR, 1.23; 95% CI, 1.09-1.38); for mood disorders including depression, 270 per 1000 persons (HR, 1.78; 95% CI, 1.68-1.89); and for anxiety, 187 per 1000 persons (HR, 1.98; 95% CI, 1.85-2.12). The rate of Parkinson disease was similar in the 2 cohorts (8 per 1000 persons; HR, 0.96; 95% CI, 0.65-1.42). The rates of the aforementioned outcomes were highest during the first year after cardiac arrest and then declined over time. Comparisons between the cohort of patients with cardiac arrest and the general population cohort showed higher rates of epilepsy, dementia, depression, and anxiety in the cardiac arrest group.

**Conclusions and Relevance:**

In this cohort study, patients discharged after cardiac arrest had an increased rate of subsequent stroke, epilepsy, dementia, depression, and anxiety compared with patients with myocardial infarction and people from the general population, with declining rates over time. These findings suggest the need for preventive strategies and close follow-up of cardiac arrest survivors.

## Key Points

**Question:**

Are cardiac arrest survivors at increased risk for stroke, epilepsy, Parkinson disease, dementia, depression, and anxiety?

**Findings:**

In this nationwide, population-based cohort study involving 250 838 adults, patients with cardiac arrest had significantly increased rates of epilepsy, dementia, depression, and anxiety compared with a cohort of patients with myocardial infarction. Furthermore, patients with cardiac arrest had a significantly increased rate of stroke, particularly within the first year after discharge, whereas the rate of Parkinson disease was similar between groups.

**Meaning:**

The findings of this study suggest that cardiac arrest survivors may have an increased rate of common neurologic and psychiatric outcomes, underscoring the need for preventive strategies and close surveillance.

## Teaser

This cohort study uses data from Danish medical registries to examine the short-term and long-term risks of common neurologic and psychiatric outcomes for patients after hospitalization for cardiac arrest.

## Introduction

Cardiac arrest is a frequent cause of death, claiming at least 300 000 lives in Europe and the United States every year.^1,2^ Thirty-day survival after out-of-hospital cardiac arrest increased from 4% in 2001 to 13% in 2014 in Denmark.^3^ Similar survival rates have been reported in other countries.^4^ Thus, the number of cardiac arrest survivors is growing, and there is an increasing need for studies investigating the long-term implications of cardiac arrest for the incidence of complicating diseases.

The brain is prone to injury even during short periods of limited blood flow. Thus, the most frequent cause of morbidity and mortality after cardiac arrest is neurologic injury.^5^ Cohort studies of long-term outcomes after cardiac arrest have reported up to 5.6-fold increases in mortality in the initial years after discharge.^6,7^ However, only a few studies have investigated mental function in survivors of cardiac arrest.^8,9,10,11^ The findings included heightened risks of cognitive deficits, anxiety, and depression. These studies were limited by small study populations or follow-up periods of less than 1 year.^8,9,10,11^ Su et al^12^ reported an increased risk of epilepsy among patients with cardiac arrest within the first 6 months after discharge. In contrast, Morris et al^13^ did not find an increased long-term risk of seizures after cardiac arrest. To our knowledge, risks for common neurologic outcomes, such as stroke, Parkinson disease, and dementia, have not been thoroughly examined previously.

To improve our understanding of neurologic and psychiatric morbidity associated with cardiac arrest, we examined the risk of ischemic and hemorrhagic stroke, epilepsy, Parkinson disease, dementia, depression, and anxiety in patients after hospital discharge for cardiac arrest compared with matched cohorts.^14,15,16,17,18^

## Methods

### Study Design 

This nationwide population-based cohort study was conducted in Denmark between January 1, 1996, and December 31, 2016, using linkable health care registries.^19^ Denmark has a current population of 5.6 million inhabitants. The health care system is tax supported, providing free and universal access to the primary and secondary health care sectors, including care for patients with cardiac arrest.^19^ A full description of the registries used for data collection is provided in the eAppendix in the Supplement. The *International Statistical Classification of Disease and Related Health Problems, Eighth Revision* (*ICD-8*) and the *International Statistical Classification of Diseases and Related Health Problems, Tenth Revision* (*ICD-10*) codes, Anatomical Therapeutic Chemical codes, and procedure codes used in the study are provided in eTables 1-3 in the Supplement. This study followed the Strengthening the Reporting of Observational Studies in Epidemiology (STROBE) reporting guideline.

This study was approved by the Danish Data Protection Agency through registration at Aarhus University. According to Danish legislation, informed consent from patients and ethics committee approval are not required for registry-based studies because patient data are deidentified.

### Participants

All patients 18 years or older with a first-time inpatient or emergency department contact resulting in a diagnosis of cardiac arrest during the 21-year follow-up period were identified through the Danish National Patient Registry (DNPR), which covers all Danish hospitals.^20^ We identified patients using both primary and secondary discharge diagnoses for cardiac arrest. By using information in the medical records as the criterion standard, the positive predictive value of cardiac arrest is approximately 94% in the DNPR,^14^ while the sensitivity is unknown. Only patients surviving to discharge were included in the analysis.

### Comparison Cohorts

We used the Danish Civil Registration System^19^ and the DNPR^20^ to form 2 comparison cohorts in which each patient with cardiac arrest was matched by sex, year of birth, and calendar period (in 5-year intervals) with up to 10 comparators. The first comparison cohort included patients with a first-time primary inpatient diagnosis of myocardial infarction who survived until discharge to sample patients with a cardiovascular risk profile. Because myocardial infarction is the most frequent cause of cardiac arrest, this allowed us to disentangle the association of complete circulatory failure from that of cardiac ischemia when calculating the long-term risk of neurologic and psychiatric outcomes. Second, to clarify complications of cardiac arrest in a population context, we sampled a cohort of individuals from the general population without previous cardiac arrest. The aim of the matching was to address confounding and provide a balanced comparison cohort.

Sampling of comparators was performed with replacement.^15^ The index date was defined as the date of hospital discharge for patients diagnosed with cardiac arrest. For members of the comparison cohorts, the index date was defined as the date of matching with a patient who had experienced a cardiac arrest. Hospital discharge for patients with cardiac arrest and those with myocardial infarct was defined as the first discharge without a subsequent admission within the next 24 hours to account for transfers between departments before final discharge. Individuals in the comparison cohorts who had a cardiac arrest during follow-up were transferred to the cardiac arrest cohort. They also continued to be followed up in the comparison cohort to avoid informative censoring.

Patients were followed up from the hospital discharge date after a cardiac arrest or the index date until the date of an outcome, death, emigration, or December 31, 2016, whichever came first. For patients with Parkinson disease and dementia, follow-up started 6 months after the hospital discharge date or index date because diagnosis of these conditions shortly after cardiac arrest is unlikely to be a consequence of the cardiac arrest. Patients with Parkinson disease or dementia during this early 6-month period were excluded from these specific analyses in all cohorts.

### Variables

#### Outcomes

All neurologic and psychiatric outcomes were ascertained based on inpatient and hospital outpatient clinic primary and secondary *ICD-8* and *ICD-10* diagnoses recorded in the DNPR and counted, starting at the time of hospital discharge. Neurologic outcomes included ischemic and hemorrhagic stroke (inpatient diagnoses only because of the low positive predictive value of outpatient diagnoses), epilepsy, Parkinson disease, and dementia. Ischemic stroke included both patients with “specified ischemic stroke” and those with “unspecified stroke.” Dementia was also assessed using data from the Danish Psychiatric Central Research Register (DPCRR).^16^ The positive predictive values of ischemic stroke, epilepsy, Parkinson disease, and dementia range between 80% and 90% in the Danish registries, and those for hemorrhagic stroke and depression range between 70% and 75%.^17,20^

Psychiatric outcomes included diagnoses of mood disorders (including depression) and anxiety disorders based on hospital diagnoses in the DNPR and DPCRR or redemption of prescriptions for antidepressants or anxiolytics based on data from the Danish National Prescription Registry.^18^

#### Covariables

From the DNPR, the DPCRR, or both of these sources, we obtained inpatient and hospital outpatient clinic diagnoses for selected comorbidities (listed in Table 1) as well as diagnoses used to calculate a modified Charlson Comorbidity Index (CCI) score for individual patients. For stratification purposes, we also assessed treatment codes for mechanical ventilation, dialysis, administration of inotropic medications, therapeutic hypothermia, and percutaneous coronary intervention. We included information from the Danish National Prescription Registry on prescriptions for selected comedications filled within 90 days before the index date. From Statistics Denmark,^21^ we obtained information on the highest completed level of education, employment status, and personal income during the year before the index date.

**Table 1.** Characteristics of Patients Surviving a Hospital Admission for First-Time Cardiac Arrest and People in the Age- and Sex-Matched Comparison Cohorts in Denmark From 1996 to 2016

Characteristic	No. (%)		
	Cardiac arrest cohort (n = 12 046)	Matched myocardial infarction cohort (n = 118 332)	Matched general population cohort (n = 120 460)
Male	8328 (69.1)	82 338 (69.6)	83 280 (69.1)
Female	3718 (30.9)	35 994 (30.4)	37 180 (30.9)
Age, y			
<60	3950 (32.8)	37 283 (31.5)	39 449 (32.7)
60-69	2868 (23.8)	28 911 (24.4)	28 888 (24.0)
70-79	2857 (23.7)	28 704 (24.3)	28 666 (23.8)
≥80	2371 (19.7)	23 434 (19.8)	23 457 (19.5)
Median (IQR)	67 (56-76)	67 (57-76)	67 (56-75)
Period			
1996-2000	1676 (13.9)	16 440 (13.9)	16 760 (13.9)
2001-2005	2162 (17.9)	21 331 (18.0)	21 620 (17.9)
2006-2010	3098 (25.7)	30 420 (25.7)	30 980 (25.7)
2011-2016	5110 (42.4)	50 141 (42.4)	51 100 (42.4)
Comorbidity			
Heart failure	3851 (32.0)	23 189 (19.6)	4547 (3.8)
Coronary artery disease	5014 (41.6)	80 624 (68.1)	11 806 (9.8)
Atrial fibrillation or flutter	3230 (26.8)	13 667 (11.5)	7175 (6.0)
Valvular heart disease	1021 (8.5)	5688 (4.8)	2585 (2.1)
Hypercholesterolemia^a^	4831 (40.1)	95 270 (80.5)	28 163 (23.4)
Hypertension^a^	8918 (74.0)	112 287 (94.9)	59 825 (49.7)
Peripheral artery disease	479 (4.0)	5362 (4.5)	1717 (1.4)
Obesity	823 (6.8)	9416 (8.0)	3396 (2.8)
Diabetes^a^	2085 (17.3)	25 294 (21.4)	11 056 (9.2)
Chronic pulmonary disease^a^	3734 (31.0)	34 900 (29.5)	25 285 (21)
Myxedema	286 (2.4)	2310 (2.0)	1220 (1.0)
Alcohol use disorder–related diseases	1057 (8.8)	4875 (4.1)	3816 (3.2)
Head trauma	557 (4.6)	4885 (4.1)	4354 (3.6)
Anemia	984 (8.2)	6735 (5.7)	3768 (3.1)
Kidney disease	914 (7.6)	4744 (4.0)	1995 (1.7)
Stroke or TIA	1445 (12.0)	9918 (8.4)	6110 (5.1)
Cancer	1584 (13.1)	12 135 (10.3)	11 883 (9.9)
Intracranial infection	99 (0.8)	661 (0.6)	506 (0.4)
Brain tumor	17 (0.1)	63 (0.1)	67 (0.1)
Alzheimer disease	89 (0.7)	878 (0.7)	1004 (0.8)
Autism	5 (0.0)	<5 (0.0)	18 (0.0)
Depression^a^	2802 (23.3)	34 523 (29.2)	21 134 (17.5)
Anxiety	2929 (24.3)	34 196 (28.9)	19 708 (16.4)
Epilepsy	469 (3.9)	2259 (1.9)	1631 (1.4)
Modified CCI score^b^			
Normal	9127 (75.8)	94 821 (80.1)	101 322 (84.1)
Moderate	1119 (9.3)	10 185 (8.6)	6692 (5.6)
Severe	1271 (10.6)	10 123 (8.6)	9962 (8.3)
Very severe	529 (4.4)	3203 (2.7)	2484 (2.1)
Comedication in the previous 90 d			
Antithrombotic agents	2589 (21.5)	50 972 (43.1)	16 933 (14.1)
Anticoagulants	1355 (11.2)	6046 (5.1)	4255 (3.5)
NSAIDs	1137 (9.4)	9450 (8.0)	10 947 (9.1)
Antipsychotic agents	415 (3.4)	2231 (1.9)	2648 (2.2)
Income^c^			
Low	3573 (29.7)	34 164 (28.9)	30 503 (25.3)
Intermediate	3312 (27.5)	33 252 (28.1)	29 441 (24.4)
High	2766 (23.0)	28 011 (23.7)	28 875 (24.0)
Very high	2382 (19.8)	22 877 (19.3)	31 514 (26.2)
Missing	13 (0.1)	28 (0.0)	127 (0.1)
Employment			
Employed	3777 (31.4)	37 786 (31.9)	47 915 (39.8)
Early retirement	441 (3.7)	4044 (3.4)	3487 (2.9)
Unemployed	2157 (17.9)	19 444 (16.4)	13 825 (11.5)
State pensioner	5632 (46.8)	56 795 (48.0)	54 745 (45.4)
Missing	39 (0.3)	263 (0.2)	488 (0.4)
Educational level			
Basic education, primary school	4622 (38.4)	47 966 (40.5)	40 574 (33.7)
Youth education, high school, or similar education	4433 (36.8)	44 484 (37.6)	45 613 (37.9)
Higher education	1914 (15.9)	15 800 (13.4)	24 050 (20.0)
Missing	1077 (8.9)	10 082 (8.5)	10 223 (8.5)
Length of stay, median (IQR), wk	1.9 (0.9-3.6)	1.0 (0.7-1.4)	NA
Kaplan-Meier all-cause mortality after hospital discharge, % (95% CI)			
At 1 y	16.0 (15.3-16.7)	4.9 (4.8-5.1)	3.1 (3.0-3.2)
At 5 y	38.4 (37.4-39.3)	22.7 (22.5-23.0)	16.0 (15.8-16.3)
At 10 y	56.8 (55.6-58.0)	42.6 (42.2-43.0)	32.0 (31.7-32.4)

Abbreviations: CCI, Charlson Comorbidity Index; NA, not applicable; NSAIDs, nonsteroidal anti-inflammatory drugs; TIA, transient ischemic attack.

^a^ Algorithms used to define comorbidity to ensure inclusion of patients diagnosed and treated in the primary sector are described in eTable 3 in the Supplement.

^b^ Categories of comorbidity were based on modified CCI scores: 0 (normal), 1 (moderate), 2 (severe), and 3 or higher (very severe). Conditions included in the CCI are described in eTable 2 in the Supplement.

^c^ Income categories are derived from yearly quartiles; thus, definitions of those quartiles changed during the years of the study period.

### Statistical Analysis

We characterized the cardiac arrest and comparison cohorts according to sex, age groups, all-cause mortality, calendar periods, and the covariables presented in Table 1. All-cause mortality was computed using Kaplan-Meier survival analysis.

We used the cumulative incidence (risk) function to calculate absolute rates of all outcomes, accounting for death as a competing risk.^22^ Hazard ratios (HRs) were computed using multivariable stratified Cox proportional hazards regression models, comparing the cardiac arrest cohort with the matched comparison cohorts. In the analyses of stroke and psychiatric outcomes, we excluded patients with an outcome within the year before their index date to ensure recovery from these past outcomes and to increase the probability of an association between new outcomes and cardiac arrest or myocardial infarction. Similarly, we excluded patients with a diagnosis of epilepsy, Parkinson disease, or dementia before their index date because we considered these to be chronic diseases. We controlled the HRs for the matching factors and adjusted them for the covariables presented in Table 1. Cumulative incidence curves were restricted to 15 years because the low number of observations thereafter would compromise data anonymity. The proportional hazards assumption was assessed graphically using log-log plots, and no overall violations were detected except for ischemic stroke. To compensate for this, we also reported stroke, epilepsy, and psychiatric estimates for 0 to 3 months, 4 to 12 months, 13 to 60 months, and more than 5 years of follow-up. These periods were defined a priori.

All statistical analyses were performed using SAS, version 9.2 (SAS Institute Inc). Statistical significance was defined as a 95% CI excluding 1.

#### Subgroup Analyses

We conducted subgroup analyses by age group, sex, calendar period, socioeconomic status, preexisting cardiac disease, length of hospital stay, and CCI score. An additional analysis was restricted to patients who had cardiac arrest with shockable cardiac rhythms because nonshockable rhythms do not have a specific *ICD-8* or *ICD-10* code. Subgroup analyses for therapeutic hypothermia, coronary angiography, percutaneous coronary intervention, need for intensive care unit (ICU) admission, mechanical ventilation, administration of inotropic medications, and dialysis were conducted among patients diagnosed starting on January 1, 2005, since these data were not available in the DNPR earlier.

#### Sensitivity Analyses

The robustness of our findings was assessed in several sensitivity analyses. To improve the specificity of the stroke diagnosis, we restricted analysis to patients who were diagnosed with stroke and underwent a computed tomographic or magnetic resonance imaging scan of the brain during the same admission. This analysis was restricted to patients diagnosed starting on January 1, 2000, when these data became available. Furthermore, we separately analyzed patients with “ischemic stroke” and “unspecified stroke.” We restricted mood disorder outcomes (including depression) to a first-time diagnosis and performed an analysis with depression outcomes only. We formed a comparison cohort consisting of patients receiving mechanical ventilation in the ICU from January 1, 2000, to further isolate the risk contributed by cardiac arrest. Patients were matched by sex, year of birth, and calendar period.

## Results

### Participants and Descriptive Data

Among 250 838 individuals involved in this study (median age, 67 years [IQR, 57-76 years]; 173 946 [69.3%] male; 76 892 [30.7%] female), 12 046 were survivors of cardiac arrest (median age, 67 years [IQR, 56-76 years]; 8328 [69.1%] male; median follow-up, 3.6 years [IQR, 1.2-7.4 years]; 1-year postdischarge mortality, 16.0%). The matched comparison cohorts included 118 332 patients with myocardial infarction (median age, 67 years [IQR, 57-76 years]; 82 338 [69.6%] male; median follow-up, 4.8 years [IQR, 2.1-8.6 years]; 1-year mortality, 4.9%) and 120 460 individuals from the general population (median age, 67 years [IQR, 56-75 years]; 83 280 [69.1%] male; median follow-up, 5.4 years [IQR, 2.5-9.6 years]; 1-year mortality, 3.1%) (Table 1).

### Risk of Neurologic Outcomes

#### Cardiac Arrest Cohort vs Myocardial Infarction Cohort

**Figure 1.**
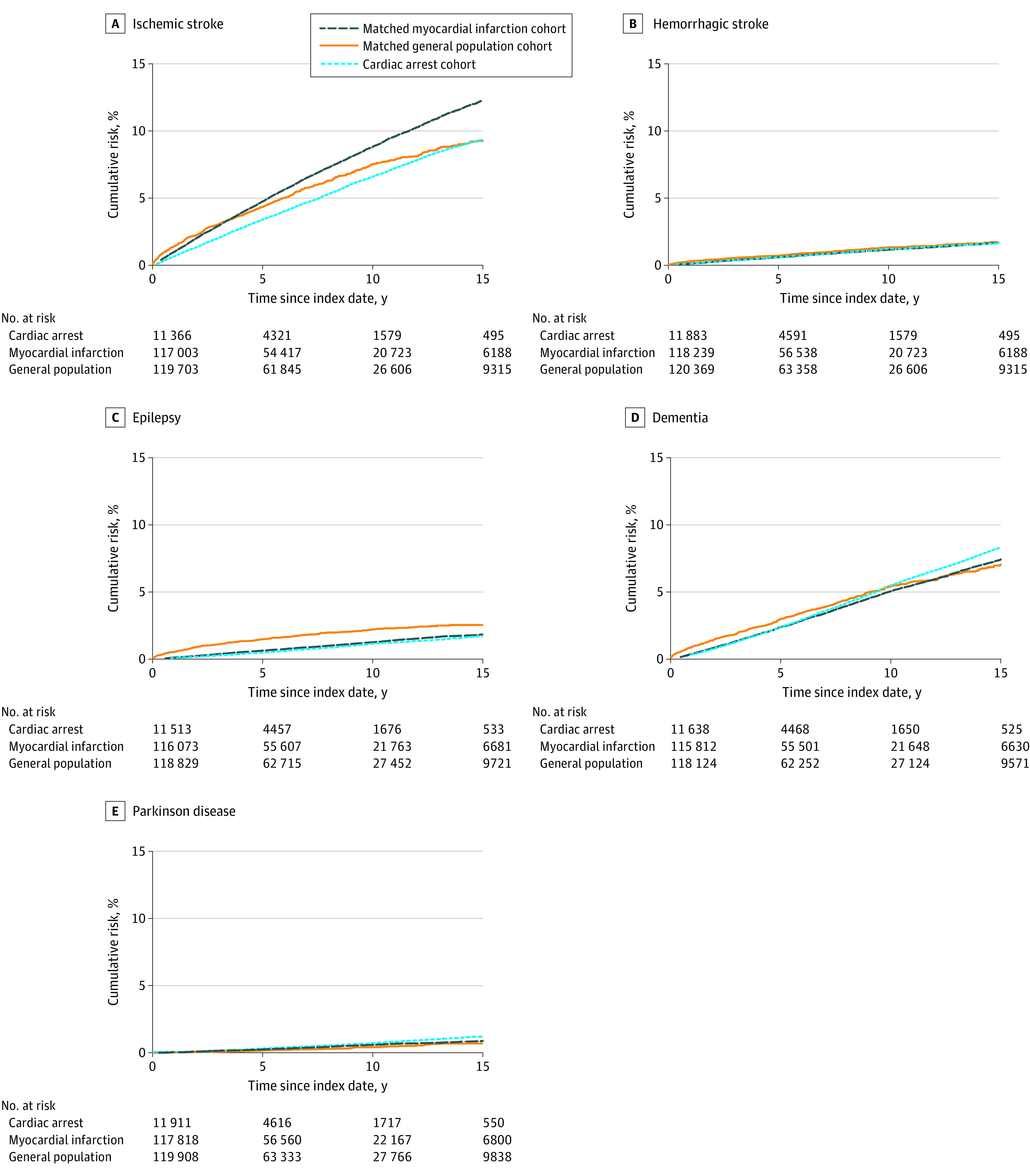
Cumulative Incidence Curves of Neurologic Outcomes in the Cardiac Arrest and Matched Comparison Cohorts Outcomes are after hospital discharge.

Cumulative incidences (risk) per 1000 persons and HRs for the study outcomes are shown in Table 2, and cumulative incidence curves are provided in Figure 1. During the 21-year follow-up period, the rates of ischemic and hemorrhagic stroke were similar in the cardiac arrest and myocardial infarction cohorts. However, when the analysis was restricted to the first year after discharge, the rates of ischemic stroke (10 per 1000 persons; HR, 1.30; 95% CI, 1.02-1.64) and hemorrhagic stroke (2 per 1000 persons; HR, 2.03; 95% CI, 1.12-3.67) were higher in the cardiac arrest cohort than in the myocardial infarction cohort. The 21-year risk of epilepsy was 28 per 1000 persons in the cardiac arrest cohort compared with 31 per 1000 persons in the myocardial infarction cohort, with an adjusted HR of 2.01 (95% CI, 1.66-2.44). The rates of stroke and epilepsy among patients with cardiac arrest were notably increased during the first year after hospital discharge but thereafter approximated those of the myocardial infarct cohort. Compared with the myocardial infarction cohort, patients in the cardiac arrest cohort had an increased risk of dementia, with a 21-year risk of 73 per 1000 persons (HR, 1.23; 95% CI, 1.09-1.38). Dementia outcomes 0 to 6 months after cardiac arrest were excluded from the analysis. Including them would strengthen the association even more (6-month risk, 6 per 1000 persons; HR, 2.98; 95% CI, 2.10-4.22). The rate of Parkinson disease was similar in the 2 cohorts: 8 per 1000 persons (HR, 0.96; 95% CI, 0.65-1.42)

**Table 2.** Risk of Neurologic Outcomes Among Patients With Cardiac Arrest and People in the Matched Comparison Cohorts

Outcome by time since discharge	Cardiac arrest cohort		Matched MI cohort		Matched general population cohort		Adjusted HR (95% CI)^a^	
	No. of outcomes/No. at risk	Cumulative incidence per 1000 persons (95% CI)	No. of outcomes/No. at risk	Cumulative incidence per 1000 persons (95% CI)	No. of outcomes/No. at risk	Cumulative incidence per 1000 persons (95% CI)	Cardiac arrest cohort vs matched MI cohort	Cardiac arrest cohort vs matched general population cohort
Ischemic stroke								
0-3 mo	61/11 366	5.41 (4.19-6.91)	298/117 003	2.58 (2.30-2.88)	167/119 703	1.41 (1.21-1.64)	1.84 (1.34-2.55)	3.49 (2.41-5.04)
4-12 mo	96/10 154	9.70 (7.92-11.79)	825/112 974	7.53 (7.03-8.05)	604/116 361	5.35 (4.94-5.79)	1.30 (1.02-1.64)	1.53 (1.19-1.97)
13-60 mo	262/8733	35.21 (31.17-39.61)	3391/101 557	39.70 (38.40-41.04)	2531/106 350	28.28 (27.21-29.39)	0.88 (0.76-1.01)	1.10 (0.95-1.27)
>5 y	236/4321	108.12 (90.84-127.07)	3455/54 417	152.92 (134.35-172.60)	2890/61 845	101.59 (96.21-107.13)	0.89 (0.76-1.04)	1.26 (1.07-1.48)
Overall	655/11 366	108.97 (97.66-120.96)	7969/117 003	162.03 (147.93-176.71)	6192/119 703	117.48 (112.92-122.14)	0.98 (0.90-1.08)	1.29 (1.18-1.42)
Hemorrhagic stroke								
0-3 mo	16/11 883	1.36 (0.82-2.18)	36/118 239	0.31 (0.22-0.42)	44/120 369	0.37 (0.27-0.49)	5.06 (2.11-12.16)	4.78 (2.20-10.39)
4-12 mo	19/10 640	1.84 (1.15-2.83)	110/114 337	0.99 (0.82-1.19)	101/117 099	0.89 (0.73-1.08)	2.03 (1.12-3.67)	1.77 (1.00-3.14)
13-60 mo	39/9182	4.93 (3.57-6.69)	432/103 232	4.96 (4.51-5.45)	446/107 375	4.91 (4.47-5.38)	0.90 (0.62-1.30)	0.95 (0.65-1.39)
>5 y	47/4591	22.17 (12.36-36.80)	488/56 538	19.22 (16.70-22.02)	520/63 358	18.35 (16.06-20.88)	0.99 (0.68-1.42)	1.52 (1.04-2.22)
Overall	121/11 883	20.77 (14.17-29.42)	1066/118 239	20.81 (18.78-22.99)	1111/120 369	21.37 (19.37-23.52)	1.13 (0.91-1.41)	1.37 (1.10-1.72)
Epilepsy								
0-3 mo	31/11 513	2.72 (1.89-3.82)	26/116 073	0.23 (0.15-0.33)	11/118 829	0.09 (0.05-0.17)	14.90 (5.70-38.98)	NR^b^
4-12 mo	29/10 316	2.88 (1.98-4.10)	97/112 270	0.89 (0.73-1.09)	74/115 624	0.66 (0.52-0.82)	2.40 (1.37-4.18)	3.35 (1.77-6.32)
13-60 mo	86/8896	11.14 (8.97-13.70)	459/101 424	5.42 (4.94-5.93)	375/106 059	4.22 (3.81-4.66)	1.88 (1.41-2.52)	2.45 (1.83-3.28)
>5 y	55/4457	21.93 (15.97-29.38)	545/55 607	31.58 (17.94-51.33)	576/62 715	20.75 (18.38-23.34)	1.35 (0.95-1.93)	1.59 (1.13-2.24)
Overall	201/11 513	28.11 (23.67-33.11)	1127/116 073	30.60 (19.65-45.33)	1036/118 829	22.24 (20.18-24.44)	2.01 (1.66-2.44)	3.14 (2.59-3.79)
Dementia								
6-60 mo	222/9896	27.25 (23.85-30.99)	1983/108 352	22.46 (21.49-23.46)	2045/111 702	22.55 (21.59-23.54)	1.23 (1.05-1.44)	1.49 (1.27-1.75)
>5 y	174/4468	65.54 (55.80-76.29)	2065/55 501	65.45 (62.50-68.48)	2530/62 252	71.00 (68.13-73.94)	1.21 (1.00-1.46)	1.26 (1.04-1.52)
Overall^c^	396/9896	72.66 (65.03-80.82)	4048/108 352	74.29 (71.77-76.87)	4575/111 702	83.02 (80.41-85.69)	1.23 (1.09-1.38)	1.38 (1.22-1.56)
Parkinson disease								
6-60 mo	13/10 124	1.65 (0.93-2.78)	207/110 184	2.33 (2.03-2.67)	268/113 386	2.95 (2.61-3.33)	0.71 (0.38-1.31)	0.86 (0.46-1.58)
>5 y	22/4616	8.43 (5.33-12.82)	263/56 560	8.19 (7.17-9.33)	373/63 333	10.77 (9.64-11.99)	1.26 (0.75-2.14)	0.79 (0.48-1.31)
Overall^c^	35/10 124	7.53 (5.16-10.68)	470/110 184	8.83 (7.96-9.79)	641/113 386	12.14 (11.11-13.24)	0.96 (0.65-1.42)	0.87 (0.59-1.26)

Abbreviations: HR, hazard ratio; MI, myocardial infarction; NR, not reported.

^a^ Controlled for age, sex, calendar year, and adjusted for the variables in Table 1.

^b^ High values result from low number of events in some covariable levels.

^c^ Presented as an overall estimate only because the first 6 months of follow-up were disregarded.

#### Cardiac Arrest Cohort vs General Population Cohort

Hazard ratios for stroke, epilepsy, and dementia were increased when the cardiac arrest cohort and general population cohort were compared, with overall adjusted HRs of 1.29 (95% CI, 1.18-1.42) for ischemic stroke, 1.37 (95% CI, 1.10-1.72) for hemorrhagic stroke, 3.14 (95% CI, 2.59-3.79) for epilepsy, and 1.38 (95% CI, 1.22-1.56) for dementia. These associations between cardiac arrest and the study outcomes exhibited a similar decline in rates over time (Table 2).

### Risk of Psychiatric Disorders

#### Cardiac Arrest Cohort vs Myocardial Infarction Cohort

**Figure 2.**
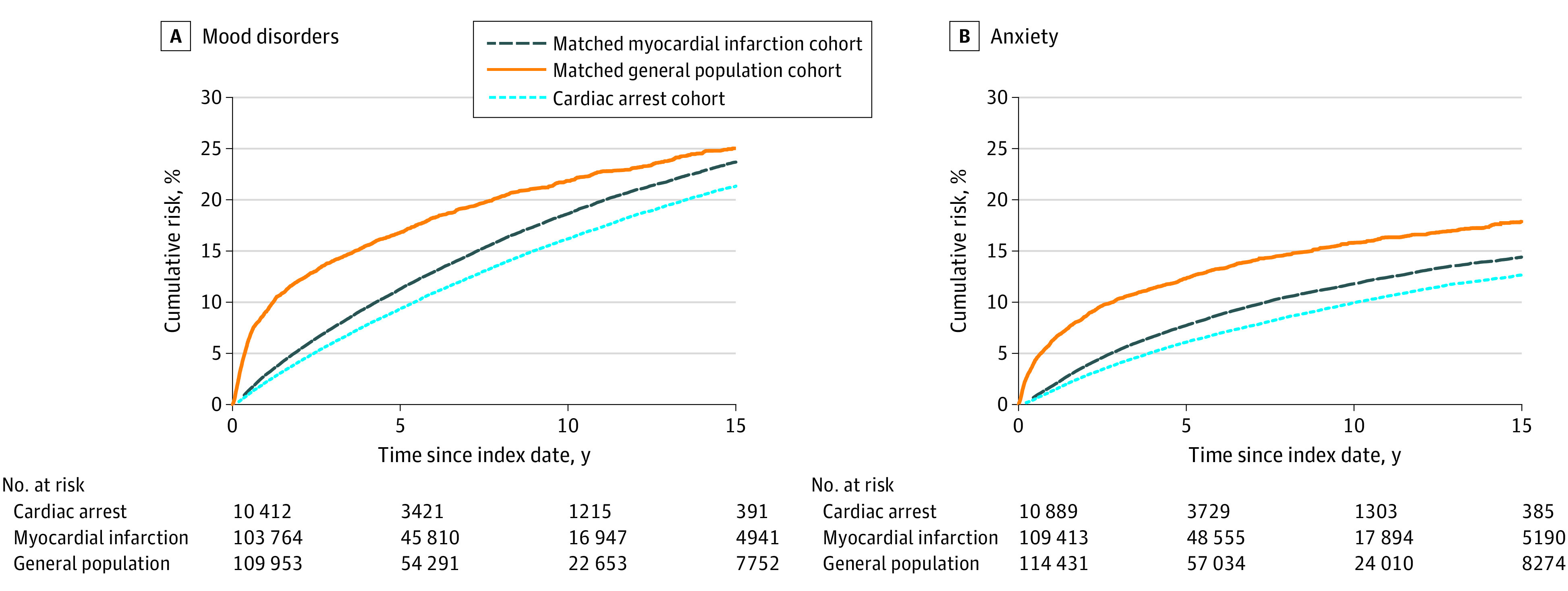
Cumulative Incidence Curves of Psychiatric Outcomes in the Cardiac Arrest and Matched Comparison Cohorts Outcomes are after hospital discharge.

Risk per 1000 persons and HRs for psychiatric outcomes are provided in Table 3, and cumulative incidence curves are presented in Figure 2. The 21-year risk of mood disorders (including depression) was 270 per 1000 persons for the cardiac arrest cohort and 296 per 1000 persons for the myocardial infarction cohort. After adjusting for comorbidity, the patients with cardiac arrest had an increased rate of mood disorders including depression compared with the patients with myocardial infarction (HR, 1.78; 95% CI, 1.68-1.89). For anxiety, the numbers were similar, with 21-year risks of 187 per 1000 persons for the cardiac arrest cohort and 167 per 1000 persons for the myocardial infarction cohort, with an adjusted HR of 1.98 (95% CI, 1.85-2.12). As for stroke and epilepsy, the increased rate was most pronounced during the first year after discharge.

**Table 3.** Risk of Psychiatric Outcomes Among Patients With Cardiac Arrest and People in the Matched Comparison Cohorts

Outcome by time since discharge	Cardiac arrest cohort		Matched MI cohort		Matched general population cohort		Adjusted HR (95% CI)^a^	
	No. of outcomes/No. at risk	Cumulative incidence per 1000 persons (95% CI)	No. of outcomes/No. at risk	Cumulative incidence per 1000 persons (95% CI)	No. of outcomes/No. at risk	Cumulative incidence per 1000 persons (95% CI)^a^	Cardiac arrest cohort vs matched MI cohort	Cardiac arrest cohort *vs* matched general population cohort
Mood disorders including depression								
0-3 mo	363/10 412	35.30 (31.86-39.00)	567/103 764	5.54 (5.10-6.01)	445/109 953	4.10 (3.74-4.50)	7.10 (5.94-8.49)	8.20 (6.80-9.90)
4-12 mo	551/9060	62.20 (57.30-67.37)	2334/100 025	24.04 (23.09-25.02)	1847/106 721	17.83 (17.04-18.65)	2.87 (2.55-3.22)	2.93 (2.60-3.31)
13-60 mo	644/7428	99.70 (92.48-107.23)	6756/88 750	89.93 (87.88-92.01)	6154/96 628	75.07 (73.27-76.91)	1.24 (1.13-1.36)	1.27 (1.15-1.40)
>5 y	346/3421	192.08 (157.72-229.05)	5105/45 810	219.44 (210.78-228.22)	5366/54 291	199.11 (191.25-207.08)	1.00 (0.87-1.14)	1.05 (0.92-1.19)
Overall	1904/10 412	269.82 (249.79-290.22)	14 762/103 764	268.96 (262.50-275.45)	13 812/109 953	250.16 (243.74-256.63)	1.78 (1.68-1.89)	1.80 (1.70-1.91)
Anxiety								
0-3 mo	289/10 889	26.84 (23.91-30.02)	348/109 413	3.23 (2.90-3.58)	236/114 431	2.09 (1.84-2.37)	9.48 (7.60-11.84)	10.44 (8.24-13.25)
4-12 mo	369/9540	39.72 (35.88-43.83)	1554/105 553	15.21 (14.47-15.97)	1231/111 159	11.44 (10.81-12.09)	2.86 (2.49-3.30)	3.09 (2.67-3.59)
13-60 mo	533/7975	76.99 (70.81-83.49)	5059/94 076	63.16 (61.48-64.87)	4262/100 928	49.66 (48.21-51.14)	1.37 (1.23-1.52)	1.36 (1.22-1.52)
>5 y	234/3729	113.60 (97.48-131.08)	2853/48 555	122.03 (115.25-129.02)	3020/57 034	113.97 (105.79-122.47)	1.24 (1.06-1.46)	1.26 (1.07-1.48)
Overall	1425/10 889	187.12 (175.98-198.53)	9814/109 413	166.70 (161.45-172.03)	8749/114 431	152.98 (146.17-159.92)	1.98 (1.85-2.12)	1.97 (1.84-2.12)

Abbreviations: HR, hazard ratio; MI, myocardial infarction.

^a^ Controlled for age, sex, calendar year, and adjusted for the variables in Table 1.

#### Cardiac Arrest Cohort vs General Population Cohort

A comparison of psychiatric outcomes in the cardiac arrest cohort and in the general population cohort yielded HRs similar to those for the cardiac arrest vs myocardial infarction cohorts, with the same decline in rate over time (Table 3). For example, mood disorders including depression (HR, 1.80; 95% CI, 1.70-1.91) and anxiety (HR, 1.97; 95% CI, 1.84-2.12) had the same decline in rate over time.

### Additional Analyses

The results of the stratified analyses were broadly consistent with the results of the main analyses presented in eTables 4 to 7 in the Supplement. Associations with higher HRs were observed for epilepsy, dementia, and psychiatric outcomes for people 60 years or younger, but the absolute risks were low. A hospital stay of more than 4 weeks, need for ICU admission, mechanical ventilation, dialysis, and administration of inotropic medications were all associated with increased rates of epilepsy and psychiatric outcomes.

The results of the sensitivity analyses were not appreciably different from the results of the main analyses (eTables 8 to 11 in the Supplement). The comparison between survivors of cardiac arrest and patients who received mechanical ventilation in the ICU showed an increased risk of psychiatric outcomes among the cardiac arrest survivors (eTable 12 in the Supplement).

## Discussion

In this nationwide cohort study, we found associations between cardiac arrest and development of neurologic and psychiatric disorders. During a 21-year follow-up period, risks of most outcomes were increased within the first year after discharge, then decreased in subsequent years, approximating unity after 5 years for comparisons with a myocardial infarction cohort. However, except for mood disorders, risks generally remaining elevated for a general population cohort.

### Comparison of Findings With Existing Literature

A systematic review of psychologic distress among cardiac arrest survivors revealed that the prevalence of anxiety and depression varied greatly among studies in the period of 1 to 72 months after discharge (anxiety, 13%-61%; depression, 14%-45%).^9^ However, the studies were small, with sample sizes ranging from 21 to 168 patients. Most studies also had follow-up periods of less than 12 months and lacked comparison cohorts. Small observational studies have also reported a high prevalence of dementia symptoms among cardiac arrest survivors,^23^ whereas the risk of Parkinson disease has not been studied in these patients, to our knowledge. In a cohort study of 1346 patients in Taiwan who experienced cardiac arrests, Su et al^12^ found an increased risk of epilepsy among cardiac arrest survivors compared with the general population (HR, 20.83; 95% CI, 12.24-35.43), especially within the first 6 months after discharge. An elevated risk of morbidity could not be confirmed by Morris et al,^13^ who reported an adjusted HR of 0.9 (95% CI, 0.9-1.0) in their study of long-term risk of seizures among cardiac arrest survivors in the US. In contrast, our population-based cohort study was large enough to examine the associations between cardiac arrest and several neurologic and psychiatric disorders based on clinical diagnoses. Our data are in line with data from a large cohort study showing an approximately 3-fold increase in stroke among cardiac arrest survivors compared with control participants from the general population.^24^

Andrew et al^6^ reported 5.6-fold increased mortality among cardiac arrest survivors the first year after discharge, followed by a declining risk approximating that of the general population after 5 years. In our study increased mortality was also observed among cardiac arrest survivors, which extends these findings with data on the longitudinal development of neurologic and psychiatric outcomes.

### Explanation of Results

The neuronal injury caused by circulatory arrest and subsequent reperfusion^25^ could be associated with increased risk for several neurologic disorders, such as the increased risk of epilepsy observed in the present study. The high short-term risk of stroke could be caused by post–cardiac arrest syndrome, including hypotension, inflammation, activated coagulation, and emboli resulting from myocardial dysfunction,^24^ combined with neuronal injury. As patients stabilize and recover clinically, no further damage to the brain occurs, and the risk of neurologic outcomes declines over time. In our study, the higher risk of stroke persisting after 3 months in the cardiac arrest cohort compared with the general population cohort could have been caused by the increased burden of disease associated with cardiac arrest (eg, atrial fibrillation and heart failure).^26,27,28^ Treatment with agents such as anticoagulants and antithrombotics may have increased the risk of hemorrhagic stroke in both the cardiac arrest cohort and the myocardial infarction cohort.^29,30^ An increased burden of cardiovascular disease combined with the initial global ischemic insult to the brain may cause dispersed neuronal injury, explaining the increased risk of dementia. More specific pathologic pathways may have been less affected by the dispersed neuronal injury, resulting in a more equal rate of Parkinson disease among the 3 cohorts.

Many patients who have been in the ICU and are survivors of a myocardial infarction experience anxiety and depression for months after discharge.^31,32,33^ Our study further explored the association between psychiatric outcomes and cardiac arrest by comparing the cardiac arrest cohort with a matched ICU cohort. The results suggest that the risk of anxiety and depression among cardiac arrest survivors is not solely explained by the ICU admission because these patients had a higher risk of these outcomes than patients who received mechanical ventilation in the ICU.

### Care for Patients With Cardiac Arrest After Discharge

Early postdischarge follow-up of the increasing number of cardiac arrest survivors may help health care professionals to recognize, diagnose, and treat potential neurologic or psychiatric complications and to provide cost-efficient care. Our data support close monitoring of cardiac arrest survivors during the initial months after discharge, similar to the post–myocardial infarct program offered in many countries.^34^ Routine follow-up can likely be reduced after the first year, with future medical consultations guided by the same clinical principles applicable to the general population. Similarly, preventive strategies are likely to have the largest impact during the first year after discharge.

### Limitations

This study has limitations. Although the positive predictive value of a cardiac arrest diagnosis in the DNPR has been validated, its completeness is unknown.^14^ However, discharged cardiac arrest survivors in Denmark total approximately 700 annually (when the numbers of 30-day survivors from 2 publications covering in-hospital and out-of-hospital cardiac arrest are combined).^35,36^ This estimate is in line with the annual number of 602 cardiac arrest cases included in our study. Hence, we have no reason to believe selection bias is a problem in our data set. The high positive predictive value of the outcome and comorbidity diagnoses in the DNPR has been demonstrated previously,^20,37^ minimizing the risk of overestimating the established associations. The sensitivity of the outcomes in the DNPR is, however, unknown, posing a risk of underestimation of the associations. We acknowledge that the lack of data on circumstances regarding the cardiac arrest is a limitation of the study; however, these data are not a part of the DNPR.

To capture patients treated primarily by general practitioners (whose records are not in the nationwide registries), filled prescriptions were included in the outcome definition for psychiatric disorders. However, psychoactive medications are not used solely for their approved indications. Thus, some degree of misclassification of depression and anxiety may have occurred. However, it is unlikely that the misclassification would have differed among the cohorts, and thus, any potential bias would have been toward the null.

## Conclusions

In this nationwide, population-based cohort study, comparisons of patients who had survived cardiac arrest with those who had experienced myocardial infarction without cardiac arrest and people from the general population demonstrated an association between cardiac arrest and neurologic and psychiatric outcomes. The risk of these outcomes was greatest within the first year after discharge. Except for mood disorders, risks declined but remained elevated when compared with the general population after 5 years. These findings suggest the need for preventive strategies and close follow-up of cardiac arrest survivors.

## Notes

Supplement. **eAppendix.** Registry Descriptions **eTable 1.** *International Classification of Diseases* (*ICD*) Codes Used in the Study **eTable 2.** Modified Charlson Comorbidity Index Conditions **eTable 3.** Algorithms Used to Identify Comorbidity to Ensure Inclusion of Patients Diagnosed and Treated in the Primary Sector **eTable 4.** Hazard Ratios for Neurological and Psychiatric Outcomes Among Patients With Cardiac Arrest Compared With Myocardial Infarction Patients, Stratified by Matching Factors, Socioeconomic Status, Length of Stay, and Comorbidity **eTable 5.** Hazard Ratios for Neurological and Psychiatric Outcomes Among Patients With Cardiac Arrest Compared With Myocardial Infarction Patients, Stratified by Comorbidity and Treatment Interventions **eTable 6.** Hazard Ratios for Neurological and Psychiatric Outcomes Among Patients With Cardiac Arrest Compared With the General Population, Stratified by Matching Factors, Socioeconomic Status, Length of Stay, and Comorbidity **eTable 7.** Hazard Ratios for Neurological and Psychiatric Outcomes Among Patients With Cardiac Arrest Compared With the General Population, Stratified by Comorbidity and Treatment Interventions **eTable 8.** Sensitivity Analyses Examining Risk of Stroke Outcomes Among Patients With Cardiac Arrest and People in the Matched Comparison Cohorts **eTable 9.** Sensitivity Analyses Examining Risk of Specified and Unspecified Ischemic Stroke Among Patients With Cardiac Arrest and People in the Matched Comparison Cohorts **eTable 10.** Sensitivity Analyses Examining Risk of First-time Diagnosis of Mood Disorders Among Patients With Cardiac Arrest and People in the Matched Comparison Cohorts **eTable 11.** Sensitivity Analysis Examining the Risk of Depression (Excluding Other Mood Disorders) Among Patients With Cardiac Arrest and People in the Matched Comparison Cohorts **eTable 12.** Overall Risk of Neurological and Psychiatric Outcomes Among Cardiac Arrest Patients and ICU Patients in the Matched Comparison Cohort

## References

[1] Gräsner JT, Herlitz J, Tjelmeland IBM, . European Resuscitation Council Guidelines 2021: epidemiology of cardiac arrest in Europe. Resuscitation. 2021;161:61-79. doi:10.1016/j.resuscitation.2021.02.007 33773833

[2] Virani SS, Alonso A, Benjamin EJ, ; American Heart Association Council on Epidemiology and Prevention Statistics Committee and Stroke Statistics Subcommittee. Heart disease and stroke statistics—2020 update: a report from the American Heart Association. Circulation. 2020;141(9):e139-e596. doi:10.1161/CIR.0000000000000757 31992061

[3] Danish Cardiac Arrest Register. Cardiac arrest outside hospital in Denmark: summary of results from Danish Cardiac Arrest Registration 2001-2014. Hjertestop uden for Hospital i Danmark: sammenfatning af resultater fra Dansk Hjertestopregistrering 2001-2014. 2014. Accessed November 28, 2022. https://genoplivning.dk/wp-content/uploads/2016/05/Rapport-fra-Dansk-Hjertestopregister-2001-2014.pdf

[4] Kiguchi T, Okubo M, Nishiyama C, . Out-of-hospital cardiac arrest across the world: first report from the International Liaison Committee on Resuscitation (ILCOR). Resuscitation. 2020;152:39-49. doi:10.1016/j.resuscitation.2020.02.044 32272235

[5] Laver S, Farrow C, Turner D, Nolan J. Mode of death after admission to an intensive care unit following cardiac arrest. Intensive Care Med. 2004;30(11):2126-2128. doi:10.1007/s00134-004-2425-z 15365608

[6] Andrew E, Nehme Z, Wolfe R, Bernard S, Smith K. Long-term survival following out-of-hospital cardiac arrest. Heart. 2017;103(14):1104-1110. doi:10.1136/heartjnl-2016-310485 28258247

[7] Lindner T, Vossius C, Mathiesen WT, Søreide E. Life years saved, standardised mortality rates and causes of death after hospital discharge in out-of-hospital cardiac arrest survivors. Resuscitation. 2014;85(5):671-675. doi:10.1016/j.resuscitation.2014.01.002 24412645

[8] Grubb NR, O’Carroll R, Cobbe SM, Sirel J, Fox KA. Chronic memory impairment after cardiac arrest outside hospital. BMJ. 1996;313(7050):143-146. doi:10.1136/bmj.313.7050.143 8688775 PMC2351568

[9] Wilder Schaaf KP, Artman LK, Peberdy MA, ; Virginia Commonwealth University ARCTIC Investigators. Anxiety, depression, and PTSD following cardiac arrest: a systematic review of the literature. Resuscitation. 2013;84(7):873-877. doi:10.1016/j.resuscitation.2012.11.021 23200996

[10] Bundgaard K, Hansen SM, Mortensen RN, . Association between bystander cardiopulmonary resuscitation and redeemed prescriptions for antidepressants and anxiolytics in out-of-hospital cardiac arrest survivors. Resuscitation. 2017;115:32-38. doi:10.1016/j.resuscitation.2017.03.032 28363819

[11] Andersson AE, Rosén H, Sunnerhagen KS. Life after cardiac arrest: a very long term follow up. Resuscitation. 2015;91:99-103. doi:10.1016/j.resuscitation.2015.01.009 25613361

[12] Su CP, Wu JH, Yang MC, . Demographics and clinical features of postresuscitation comorbidities in long-term survivors of out-of-hospital cardiac arrest: a national follow-up study. Biomed Res Int. 2017;2017(4):9259182. doi:10.1155/2017/9259182 28286775 PMC5327773

[13] Morris NA, May TL, Motta M, Agarwal S, Kamel H. Long-term risk of seizures among cardiac arrest survivors. Resuscitation. 2018;129:94-96. doi:10.1016/j.resuscitation.2018.06.019 29932947

[14] Sundbøll J, Adelborg K, Munch T, . Positive predictive value of cardiovascular diagnoses in the Danish National Patient Registry: a validation study. BMJ Open. 2016;6(11):e012832. doi:10.1136/bmjopen-2016-012832 27864249 PMC5129042

[15] Heide-Jørgensen U, Adelborg K, Kahlert J, Sørensen HT, Pedersen L. Sampling strategies for selecting general population comparison cohorts. Clin Epidemiol. 2018;10:1325-1337. doi:10.2147/CLEP.S164456 30310326 PMC6165733

[16] Mors O, Perto GP, Mortensen PB. The Danish Psychiatric Central Research Register. Scand J Public Health. 2011;39(7)(suppl):54-57. doi:10.1177/1403494810395825 21775352

[17] Bock C, Bukh JD, Vinberg M, Gether U, Kessing LV. Validity of the diagnosis of a single depressive episode in a case register. Clin Pract Epidemiol Ment Health. 2009;5:4. doi:10.1186/1745-0179-5-4 19216741 PMC2660321

[18] Pottegård A, Schmidt SAJ, Wallach-Kildemoes H, Sørensen HT, Hallas J, Schmidt M. Data resource profile: the Danish National Prescription Registry. Int J Epidemiol. 2017;46(3):798-798f. 27789670 10.1093/ije/dyw213 PMC5837522

[19] Schmidt M, Pedersen L, Sørensen HT. The Danish Civil Registration System as a tool in epidemiology. Eur J Epidemiol. 2014;29(8):541-549. doi:10.1007/s10654-014-9930-3 24965263

[20] Schmidt M, Schmidt SAJ, Sandegaard JL, Ehrenstein V, Pedersen L, Sørensen HT. The Danish National Patient Registry: a review of content, data quality, and research potential. Clin Epidemiol. 2015;7:449-490. doi:10.2147/CLEP.S91125 26604824 PMC4655913

[21] Petersson F, Baadsgaard M, Thygesen LC. Danish registers on personal labour market affiliation. Scand J Public Health. 2011;39(7)(suppl):95-98. doi:10.1177/1403494811408483 21775363

[22] Austin PC, Lee DS, Fine JP. Introduction to the analysis of survival data in the presence of competing risks. Circulation. 2016;133(6):601-609. doi:10.1161/CIRCULATIONAHA.115.017719 26858290 PMC4741409

[23] Buanes EA, Gramstad A, Søvig KK, . Cognitive function and health-related quality of life four years after cardiac arrest. Resuscitation. 2015;89:13-18. doi:10.1016/j.resuscitation.2014.12.021 25596374

[24] Neumar RW, Nolan JP, Adrie C, . Post-cardiac arrest syndrome: epidemiology, pathophysiology, treatment, and prognostication: a consensus statement from the International Liaison Committee on Resuscitation (American Heart Association, Australian and New Zealand Council on Resuscitation, European Resuscitation Council, Heart and Stroke Foundation of Canada, InterAmerican Heart Foundation, Resuscitation Council of Asia, and the Resuscitation Council of Southern Africa); the American Heart Association Emergency Cardiovascular Care Committee; the Council on Cardiovascular Surgery and Anesthesia; the Council on Cardiopulmonary, Perioperative, and Critical Care; the Council on Clinical Cardiology; and the Stroke Council. Circulation. 2008;118(23):2452-2483. doi:10.1161/CIRCULATIONAHA.108.190652 18948368

[25] Björklund E, Lindberg E, Rundgren M, Cronberg T, Friberg H, Englund E. Ischaemic brain damage after cardiac arrest and induced hypothermia—a systematic description of selective eosinophilic neuronal death: a neuropathologic study of 23 patients. Resuscitation. 2014;85(4):527-532. doi:10.1016/j.resuscitation.2013.11.022 24321320

[26] Adelborg K, Szépligeti S, Sundbøll J, . Risk of stroke in patients with heart failure: a population-based 30-year cohort study. Stroke. 2017;48(5):1161-1168. doi:10.1161/STROKEAHA.116.016022 28377383

[27] Thomsen JH, Hassager C, Erlinge D, . Atrial fibrillation following out-of-hospital cardiac arrest and targeted temperature management—are we giving it the attention it deserves? Crit Care Med. 2016;44(12):2215-2222. doi:10.1097/CCM.0000000000001958 27513534

[28] Byrne C, Pareek M, Krogager ML, . Increased 5-year risk of stroke, atrial fibrillation, acute coronary syndrome, and heart failure in out-of-hospital cardiac arrest survivors compared with population controls: a nationwide registry-based study. Resuscitation. 2021;169:53-59. doi:10.1016/j.resuscitation.2021.10.024 34695442

[29] Fang MC, Go AS, Chang Y, . Death and disability from warfarin-associated intracranial and extracranial hemorrhages. Am J Med. 2007;120(8):700-705. doi:10.1016/j.amjmed.2006.07.034 17679129 PMC3534961

[30] Connolly SJ, Pogue J, Hart RG, ; ACTIVE Investigators. Effect of clopidogrel added to aspirin in patients with atrial fibrillation. N Engl J Med. 2009;360(20):2066-2078. doi:10.1056/NEJMoa0901301 19336502

[31] Hatch R, Young D, Barber V, Griffiths J, Harrison DA, Watkinson P. Anxiety, depression and post traumatic stress disorder after critical illness: a UK-wide prospective cohort study. Crit Care. 2018;22(1):310. doi:10.1186/s13054-018-2223-6 30466485 PMC6251214

[32] Wunsch H, Christiansen CF, Johansen MB, . Psychiatric diagnoses and psychoactive medication use among nonsurgical critically ill patients receiving mechanical ventilation. JAMA. 2014;311(11):1133-1142. doi:10.1001/jama.2014.2137 24643603

[33] Thombs BD, Bass EB, Ford DE, . Prevalence of depression in survivors of acute myocardial infarction. J Gen Intern Med. 2006;21(1):30-38. doi:10.1111/j.1525-1497.2005.00269.x 16423120 PMC1484630

[34] Thompson DR, Lewin RJP. Coronary disease. Management of the post-myocardial infarction patient: rehabilitation and cardiac neurosis. Heart. 2000;84(1):101-105. doi:10.1136/heart.84.1.101 10862600 PMC1729391

[35] Andersen LW, Holmberg MJ, Løfgren B, Kirkegaard H, Granfeldt A. Adult in-hospital cardiac arrest in Denmark. Resuscitation. 2019;140:31-36. doi:10.1016/j.resuscitation.2019.04.046 31075290

[36] Granfeldt A, Wissenberg M, Hansen SM, . Clinical predictors of shockable versus non-shockable rhythms in patients with out-of-hospital cardiac arrest. Resuscitation. 2016;108:40-47. doi:10.1016/j.resuscitation.2016.08.024 27616581

[37] Thygesen SK, Christiansen CF, Christensen S, Lash TL, Sørensen HT. The predictive value of ICD-10 diagnostic coding used to assess Charlson comorbidity index conditions in the population-based Danish National Registry of Patients. BMC Med Res Methodol. 2011;11:83. doi:10.1186/1471-2288-11-83 21619668 PMC3125388

