# Supplementary material for: Evaluation of Neurologic and Psychiatric Outcomes After Hospital Discharge Among Adult Survivors of Cardiac Arrest
Source: JAMA Netw Open. 2022 May 31;5(5):e2213546. doi: 10.1001/jamanetworkopen.2022.13546 (PMC9157268; doi:10.1001/jamanetworkopen.2022.13546)
Supplement: Supplement. — eAppendix. Registry Descriptions eTable 1. International Classification of Diseases (ICD) Codes Used in the Study eTable 2. Modified Charlson Comorbidity Index Conditions eTable 3. Algorithms Used to Identify Comorbidity to Ensure Inclusion of Patients Diagnosed and Treated in the Primary Sector eTable 4. Hazard Ratios for Neurological and Psychiatric Outcomes Among Patients With Cardiac Arrest Compared With Myocardial Infarction Patients, Stratified by Matching Factors, Socioeconomic Status, Length of Stay, and Comorbidity eTable 5. Hazard Ratios for Neurological and Psychiatric Outcomes Among Patients With Cardiac Arrest Compared With Myocardial Infarction Patients, Stratified by Comorbidity and Treatment Interventions eTable 6. Hazard Ratios for Neurological and Psychiatric Outcomes Among Patients With Cardiac Arrest Compared With the General Population, Stratified by Matching Factors, Socioeconomic Status, Length of Stay, and Comorbidity eTable 7. Hazard Ratios for Neurological and Psychiatric Outcomes Among Patients With Cardiac Arrest Compared With the General Population, Stratified by Comorbidity and Treatment Interventions eTable 8. Sensitivity Analyses Examining Risk of Stroke Outcomes Among Patients With Cardiac Arrest and People in the Matched Comparison Cohorts eTable 9. Sensitivity Analyses Examining Risk of Specified and Unspecified Ischemic Stroke Among Patients With Cardiac Arrest and People in the Matched Comparison Cohorts eTable 10. Sensitivity Analyses Examining Risk of First-time Diagnosis of Mood Disorders Among Patients With Cardiac Arrest and People in the Matched Comparison Cohorts eTable 11. Sensitivity Analysis Examining the Risk of Depression (Excluding Other Mood Disorders) Among Patients With Cardiac Arrest and People in the Matched Comparison Cohorts eTable 12. Overall Risk of Neurological and Psychiatric Outcomes Among Cardiac Arrest Patients and ICU Patients in the Matched Comparison Cohort [file jamanetwopen-e2213546-s001.pdf]

# Supplementary Online Content

Secher N, Adelborg K, Szentkúti P, et al. Evaluation of neurologic and psychiatric outcomes after hospital discharge among adult survivors of cardiac arrest. *JAMA Netw Open*. 2022;5(5):e2213546. doi:10.1001/jamanetworkopen.2022.13546

**eAppendix.** Registry Descriptions

**eTable 1.** *International Classification of Diseases* (*ICD*) Codes Used in the Study

**eTable 2.** Modified Charlson Comorbidity Index Conditions

**eTable 3.** Algorithms Used to Identify Comorbidity to Ensure Inclusion of Patients Diagnosed and Treated in the Primary Sector

**eTable 4.** Hazard Ratios for Neurological and Psychiatric Outcomes Among Patients With Cardiac Arrest Compared With Myocardial Infarction Patients, Stratified by Matching Factors, Socioeconomic Status, Length of Stay, and Comorbidity

**eTable 5.** Hazard Ratios for Neurological and Psychiatric Outcomes Among Patients With Cardiac Arrest Compared With Myocardial Infarction Patients, Stratified by Comorbidity and Treatment Interventions

**eTable 6.** Hazard Ratios for Neurological and Psychiatric Outcomes Among Patients With Cardiac Arrest Compared With the General Population, Stratified by Matching Factors, Socioeconomic Status, Length of Stay, and Comorbidity

**eTable 7.** Hazard Ratios for Neurological and Psychiatric Outcomes Among Patients With Cardiac Arrest Compared With the General Population, Stratified by Comorbidity and Treatment Interventions

**eTable 8.** Sensitivity Analyses Examining Risk of Stroke Outcomes Among Patients With Cardiac Arrest and People in the Matched Comparison Cohorts

**eTable 9.** Sensitivity Analyses Examining Risk of Specified and Unspecified Ischemic Stroke Among Patients With Cardiac Arrest and People in the Matched Comparison Cohorts

**eTable 10.** Sensitivity Analyses Examining Risk of First-time Diagnosis of Mood Disorders Among Patients With Cardiac Arrest and People in the Matched Comparison Cohorts

**eTable 11.** Sensitivity Analysis Examining the Risk of Depression (Excluding Other Mood Disorders) Among Patients With Cardiac Arrest and People in the Matched Comparison Cohorts

**eTable 12.** Overall Risk of Neurological and Psychiatric Outcomes Among Cardiac Arrest Patients and ICU Patients in the Matched Comparison Cohort

This supplementary material has been provided by the authors to give readers additional information about their work.

© 2022 Secher N et al. *JAMA Network Open*.

**eAppendix.** Registry Descriptions

Danish National Patient Registry (DNPR)
Contains data on all residents admitted to Danish non-psychiatric hospitals since 1977 and all contacts with hospital outpatient clinics and emergency room departments since 1995. Each admission is coded with one primary diagnosis and one or more secondary diagnoses classified according to the *International Classification of Diseases, Eighth Revision* (ICD-8) until the end of 1993 and *Tenth Revision* (ICD-10) thereafter.

Danish Civil Registration System
Contains individual-level information on all people residing in Denmark since 1968. A unique ten-digit Civil Personal Register number assigned to all people in the Danish Civil Resistration System allows for individual-level record linkage of Danish registers. Daily updated information on migration and vital status allows for nationwide cohort studies with virtually complete long-term follow-up of emigration and death.

Danish Psychiatric Central Research Register (DPCRR)
Contains data on all patients admitted to psychiatric hospitals and psychiatric wards in general hospitals in Denmark since 1970. Information on all psychiatric outpatient clinic contacts was added to the registry in 1995. Information on diagnoses is classified according to the *International Classification of Diseases, Eighth Revision* (ICD-8) until the end of 1993 and *Tenth Revision* (ICD-10) thereafter.

Danish National Prescription Registry (DNPrR)
Contains data on all redeemed prescriptions, including package size, strength, form, and Anatomical Therapeutic Chemical code, since 1995.

Statistics Denmark
Governmental organization responsible for producing statistics on a range of domestic affairs related to the Danish society with annual update since 1980. Included are statistics on education and labour market related matters.

© 2022 Secher N et al. *JAMA Network Open*.

| **eTable 1.** *International Classification of Diseases* (*ICD*) codes used in the study. | | | |
|---|---|---|---|
| | ICD-8 codes | ICD-10 codes | Procedure or ATC code |
| Cardiac arrest | 429.97, 427.27 | I46, I490 | |
| Shockable | | I490 | |
| **Covariables** | | | |
| *Cardiovascular diseases* | | | |
| Heart failure | 427.09, 427.10, 427.11, 427.19, 428.99,782.49 | I50, I11.0, I13.0, I13.2 | |
| Coronary artery disease | 413, 411 | I20, I251, I259 | |
| Atrial fibrillation or flutter | 427.93, 427.94 | I48 | |
| Valvular heart disease | 394-398 | I05, I06, I07, I08, I09.8, I34-I37, I39.0-I39.4, I39.3, I51.1A, Q22 | |
| Hypercholesterolemia | 272.00 | E780 | C10AA, C10B |
| Hypertension | 400-404 | DI10-DI15, I67.4 | C03, C07, C08, C09A-D |
| Peripheral artery disease | 443.89–443.99 | I73.9 | |
| *Non-cardiovascular diseases* | | | |
| Obesity | 277 | E65, E66, E68 | |
| Diabetes mellitus | 249, 250 (excluding 249.02, 250.02) | E10 (excluding E10.2), E11 (excluding E11.2), E14, H36.0 | A10 |
| Chronic pulmonary disease | 490-493; 515-518 | J40-J47; J60-J67; J68.4; J70.1; J70.3; J84.1; J92.0; J96.1; J98.2; J98.3 | R03A, R03BA, R03BB |
| Myxedema | 244 | E00-E03, E890 | |
| Alcoholism-related diseases | 980, 291.09-291.99, 303.09-303.99, 571.09-571.11, 577.10 | F10 (except F10.0 and F10.5), G31.2, G62.1, G72.1, I 42.6, K29.2, K70, K86.0, Z72.1 | |
| Head trauma | 800-803, 850-854, 873 | S00-S09 (excluding S00, S03 and S05) | |
| Anemia | 280-281, 283-285 | D50-55, D59, D61-D64 | |
| Chronic kidney disease | 249.02, 250.02, 753.10-753.19, 582-584, 590.09, 593.20, 792 | E102, E112, E142, N03, N05, N110, N14, N16, N18-N19, N269, Q611-Q614 | |
| Cancer | 140-199 (excluding 191) | DC00-96 (excluding DC71) | |
| Intracranial infection | 013, 036, 040-046, 052.01, 053.02, 054.03, 055.01, 056.01, 065.99 | DA17, DA390, DA80-DA89 DG00-DG09 | |
| Brain tumor | 191 | DC71 | |
| Autism | | DF840-841 | |
| *Comedications in past 90 days* | | | |
| Antithrombotics | | | B01AC |
| Anticoagulants | | | B01AA-AB and B01AD-AF |
| NSAIDs | | | M01A |
| Antipsycotics | | | N05A |
| **Outcomes** | | | |
| *Stroke* | | | |
| Ischemic stroke | 433-434 | I63-I64 | |
| Specified ischemic stroke | 433-434 | I63 | |
| Intracerebral hemorrhage | 431 | I61 | |
| Subarachnoid hemorrhage | 430 | I60 | |
| Epilepsy | 345 | G40-41 | |
| Parkinson’s disease | 342 | G20 | |

© 2022 Secher N et al. *JAMA Network Open*.

| | | | |
|---|---|---|---|
| Dementia | 094.19, 290.09, 290.10, 290.11, 290.18, 290.19, 292.09, 293.09, 293.19 | F00 series; F01 series (includes F01.0x, F01.1x, F01.2x, F01.3x, F01.8x, & F01.9x); F02 series; F03 series; F1x.73 series (F10.73 through F19.73); G23.1; G30 series; G31.0, G31.1, G31.8B, G31.8E, G31.85 | |
| Mood disorders including depressive disorders | 296.x9 (excluding 296.89), 298.09, 298.19, 300.49, 301.19 | F3x | Prescription for antidepressants N06A |
| Anxiety | 300 | F4x | Prescription for anxiolytics N05BA |
| **Procedures during admission** | | | |
| Computed tomography (CT) brain scan | | | UXCA00 |
| Magnetic resonance imaging (MRI) of brain | | | UXMA00 |
| Therapeutic hypothermia | | | BMFL38A |
| Coronary angiography | | | UXAC85 |
| Percutaneous coronary intervention | | | KFNG02, KFNG05, KFNG10 |
| ICU admission | | | NABB, NABE |
| Ventilator treatment | | | BGDA0 |
| Inotropics | | | BFHC92, BFHC93A-93E, BFHC95 |
| Dialysis | | | BJFD00, BJFD02 |

© 2022 Secher N et al. *JAMA Network Open*.

**eTable 2.** Modified Charlson Comorbidity Index conditions.

| Disease | Weight | ICD codes |
|---|---|---|
| Connective tissue disease | 1 | ICD-8: 712, 716, 734, 446, 135.99; ICD-10: M05, M06, M08, M09, M30, M31, M32, M33, M34, M35, M36, D86 |
| Ulcer disease | 1 | ICD-8: 530.91, 530.98, 531-534; ICD-10: K22.1, K25-K28 |
| Mild liver disease | 1 | ICD-8: 571, 57301, 57304; ICD-10:B18, K70.0-K70.3, K70.9, K71, K73, K74, K76.0 |
| Hemiplegia | 2 | ICD-8: 344; ICD-10: G81, G82 |
| Non-metastatic solid tumor | 2 | ICD-8: 140-194; ICD-10: C00-C75 |
| Leukemia | 2 | ICD-8: 204-207; ICD-10: C91-C95 |
| Lymphoma | 2 | ICD-8: 200-203, 275.59; ICD-10: C81-C85, C88, C90, C96 |
| Moderate to severe liver disease | 3 | ICD-8: 070.00, 070.02, 070.04, 070.06, 070.08, 573.00, 456.00-456.09; ICD-10: B15.0, B16.0, B16.2, B19.0, K70.4, K72, K76.6, I85 |
| Metastatic cancer | 6 | ICD-8: 195-198, 199; ICD-10: C76-C80 |
| AIDS | 6 | ICD-8: 079.83; ICD-10: B21-B24 |

© 2022 Secher N et al. *JAMA Network Open*.

**eTable 3.** Algorithms used to identify comorbidity to ensure inclusion of patients diagnosed and treated in the primary sector.

| | |
|---|---|
| Hypercholesterolemia | Diagnosis in the DNPR or at least 1 filled prescription for statins |
| Hypertension | Diagnosis in the DNPR or at least 1 filled prescription for antihypertensive medication |
| Diabetes mellitus | Diagnosis in the DNPR or at least 1 filled prescription for antidiabetics |
| Chronic pulmonary disease | Diagnosis in the DNPR or at least 1 filled prescription for inhaled $\beta_2$antagonists, anticholinergics, or steroids |
| Mood disorders (including depression) | Diagnosis in DNPR or DPCRR, or at least 2 filled prescriptions for antidepressives |
| Anxiety | Diagnosis in DNPR or DPCRR, or at least 2 filled prescriptions for anxiolytics |

Abbreviations: DNPR, Danish National Patient Registry. DPCRR, Danish Psychiatric Central Research Register

© 2022 Secher N et al. *JAMA Network Open*.

**eTable 4.** Hazard ratios for neurological and psychiatric outcomes among patients with cardiac arrest compared with myocardial infarction patients, stratified by matching factors, socioeconomic status, length of stay, and comorbidity.

| | Cardiac arrest cohort *vs* matched MI cohort. Adjusted overall hazard ratio (95% CI) | | | | | |
|---|---|---|---|---|---|---|
| | Ischemic stroke | Hemorrhagic stroke | Epilepsy | All-cause dementia | Mood disorders (incl. depression) | Anxiety |
| **Age, years** | | | | | | |
| <60 | 0.98 (0.79–1.20 ) | 1.31 (0.83–2.05 ) | 2.73 (1.92–3.88 ) | 3.83 (2.17–6.73 ) | 2.82 (2.52–3.17 ) | 3.40 (2.97–3.90 ) |
| 60-69 | 0.98 (0.81–1.19 ) | 0.78 (0.47–1.29 ) | 1.28 (0.85–1.91 ) | 1.33 (0.97–1.84 ) | 2.46 (2.16–2.80 ) | 2.45 (2.11–2.84 ) |
| 70-79 | 1.14 (0.97–1.35 ) | 1.08 (0.72–1.64 ) | 1.82 (1.21–2.73 ) | 1.19 (0.99–1.44 ) | 1.93 (1.71–2.17 ) | 1.84 (1.59–2.12 ) |
| ≥80 | 0.93 (0.77–1.11 ) | 1.33 (0.81–2.18 ) | 2.20 (1.30–3.72 ) | 1.05 (0.85–1.29 ) | 1.50 (1.30–1.73 ) | 1.69 (1.43–2.02 ) |
| **Sex** | | | | | | |
| Male | 1.08 (0.93–1.27 ) | 1.09 (0.74–1.58 ) | 2.83 (1.98–4.07 ) | 1.31 (1.09–1.59 ) | 2.18 (1.97–2.42 ) | 2.22 (1.97–2.50 ) |
| Female | 0.96 (0.86–1.08 ) | 1.19 (0.91–1.56 ) | 1.80 (1.42–2.27 ) | 1.18 (1.01–1.39 ) | 2.16 (2.00–2.33 ) | 2.41 (2.20–2.64 ) |
| **Calendar period** | | | | | | |
| 1996-2005 | 1.00 (0.88–1.13 ) | 0.93 (0.67–1.28 ) | 1.58 (1.15–2.15 ) | 1.29 (1.09–1.52 ) | 1.87 (1.71–2.04 ) | 2.13 (1.92–2.36 ) |
| 2006-2016 | 1.02 (0.89–1.17 ) | 1.39 (1.02–1.90 ) | 2.50 (1.93–3.24 ) | 1.18 (0.98–1.42 ) | 2.56 (2.34–2.79 ) | 2.68 (2.42–2.98 ) |
| **Income** | | | | | | |
| Low | 0.98 (0.85–1.12 ) | 1.08 (0.75–1.55 ) | 2.03 (1.50–2.74 ) | 1.10 (0.93–1.31 ) | 1.65 (1.50–1.81 ) | 1.81 (1.63–2.01 ) |
| Intermediate | 1.09 (0.94–1.27 ) | 0.86 (0.59–1.27 ) | 1.62 (1.18–2.23 ) | 1.26 (1.04–1.52 ) | 1.66 (1.50–1.83 ) | 1.71 (1.52–1.93 ) |
| High | 0.83 (0.68–1.02 ) | 1.62 (1.07–2.44 ) | 1.80 (1.25–2.60 ) | 1.21 (0.92–1.59 ) | 2.09 (1.87–2.35 ) | 2.09 (1.82–2.40 ) |
| Very high | 0.87 (0.68–1.12 ) | 1.01 (0.58–1.74 ) | 2.29 (1.50–3.49 ) | 1.34 (0.91–1.98 ) | 2.14 (1.86–2.46 ) | 2.54 (2.15–3.00 ) |
| **Length of stay, weeks** | | | | | | |
| <1 | 0.95 (0.81–1.12 ) | 0.96 (0.63–1.46 ) | 1.48 (1.07–2.04 ) | 1.17 (0.96–1.43 ) | 1.45 (1.31–1.61 ) | 1.76 (1.57–1.97 ) |
| 1-2 | 0.80 (0.68–0.94 ) | 1.14 (0.79–1.64 ) | 1.29 (0.86–1.94 ) | 0.76 (0.60–0.96 ) | 1.23 (1.10–1.37 ) | 1.56 (1.38–1.76 ) |
| 2-3 | 0.87 (0.71–1.07 ) | 0.97 (0.58–1.61 ) | 1.49 (0.94–2.36 ) | 0.78 (0.58–1.04 ) | 1.38 (1.21–1.59 ) | 1.57 (1.34–1.85 ) |
| 3-4 | 0.84 (0.60–1.19 ) | 1.14 (0.47–2.79 ) | 3.07 (1.34–7.02 ) | 0.68 (0.43–1.06 ) | 1.37 (1.12–1.69 ) | 1.42 (1.10–1.83 ) |
| ≥4 | 0.95 (0.75–1.22 ) | 1.61 (0.86–2.98 ) | 3.15 (1.85–5.37 ) | 1.55 (1.14–2.12 ) | 3.01 (2.57–3.53 ) | 2.28 (1.88–2.77 ) |
| **CCI score** | | | | | | |
| Normal | 0.96 (0.87–1.06 ) | 1.07 (0.84–1.36 ) | 1.87 (1.54–2.28 ) | 1.14 (1.00–1.29 ) | 1.74 (1.63–1.84 ) | 1.95 (1.81–2.09 ) |
| Moderate | 0.93 (0.70–1.24 ) | 1.43 (0.80–2.53 ) | 2.55 (1.53–4.25 ) | 1.71 (1.24–2.37 ) | 2.00 (1.70–2.37 ) | 1.91 (1.56–2.34 ) |
| Severe | 1.06 (0.81–1.39 ) | 1.13 (0.60–2.13 ) | 2.08 (1.20–3.59 ) | 1.18 (0.82–1.69 ) | 1.86 (1.56–2.21 ) | 1.93 (1.60–2.34 ) |
| Very severe | 1.05 (0.66–1.67 ) | 1.50 (0.57–3.97 ) | 1.42 (0.54–3.75 ) | 1.40 (0.81–2.42 ) | 1.81 (1.35–2.44 ) | 1.34 (0.97–1.86 ) |
| **Education** | | | | | | |
| Basic education, primary school | 1.02 (0.85–1.22 ) | 1.38 (0.90–2.10 ) | 2.34 (1.67–3.28 ) | 1.19 (0.94–1.51 ) | 2.00 (1.79–2.24 ) | 2.18 (1.91–2.48 ) |
| Youth education, high school, or similar education | 0.98 (0.81–1.20 ) | 1.31 (0.83–2.06 ) | 2.09 (1.47–2.98 ) | 1.20 (0.91–1.59 ) | 2.35 (2.09–2.64 ) | 2.38 (2.07–2.74 ) |
| Higher education | 0.93 (0.66–1.31 ) | 0.66 (0.30–1.45 ) | 1.36 (0.82–2.27 ) | 1.22 (0.76–1.96 ) | 1.79 (1.47–2.17 ) | 1.96 (1.55–2.47 ) |
| Missing | 1.28 (0.84–1.94 ) | 2.15 (0.66–7.07 ) | 2.29 (0.69–7.60 ) | 1.08 (0.65–1.79 ) | 1.86 (1.36–2.56 ) | 2.20 (1.57–3.08 ) |

The original matching was maintained for age, sex, and calendar period. For income, length of stay, CCI score, and education, the original matching was dissolved and an adjusted Cox regression analysis was performed for each stratum. Abbreviation: CCI, Charlson Comorbidity Index

© 2022 Secher N et al. *JAMA Network Open*.

**eTable 5.** Hazard ratios for neurological and psychiatric outcomes among patients with cardiac arrest compared with myocardial infarction patients, stratified by comorbidity and treatment interventions.

| | Cardiac arrest cohort *vs* matched MI cohort. Adjusted overall hazard ratio (95% CI) | | | | | |
|---|---|---|---|---|---|---|
| | Ischemic stroke | Hemorrhagic stroke | Epilepsy | All-cause dementia | Mood disorders (incl depression) | Anxiety |
| **Shockable cardiac rhythm** | | | | | | |
| Yes | 0.95 (0.83–1.09 ) | 1.05 (0.73–1.49 ) | 1.21 (0.86–1.70 ) | 1.12 (0.93–1.34 ) | 1.87 (1.70–2.05 ) | 2.22 (1.99–2.48 ) |
| **Preexsisting cardiac disease[a]** | | | | | | |
| Yes | 1.05 (0.95–1.16 ) | 1.18 (0.92–1.50 ) | 1.63 (1.31–2.02 ) | 1.18 (1.03–1.35 ) | 1.63 (1.53–1.74 ) | 1.86 (1.72–2.01 ) |
| No | 0.97 (0.83–1.14 ) | 1.41 (0.97–2.03 ) | 2.62 (1.98–3.46 ) | 1.34 (1.11–1.62 ) | 2.07 (1.89–2.26 ) | 2.09 (1.88–2.32 ) |
| **Preexisting chronic pulmonary disease** | | | | | | |
| Yes | 1.03 (0.84–1.25 ) | 1.24 (0.75–2.05 ) | 2.25 (1.58–3.20 ) | 1.21 (0.91–1.59 ) | 1.99 (1.76–2.24 ) | 2.03 (1.77–2.34 ) |
| No | 1.00 (0.86–1.16 ) | 1.30 (0.93–1.82 ) | 2.04 (1.55–2.67 ) | 1.14 (0.94–1.39 ) | 2.10 (1.92–2.30 ) | 2.28 (2.05–2.55 ) |
| **Therapeutic hypothermia** | | | | | | |
| Yes | 1.13 (0.73–1.74 ) | 3.00 (1.14–7.91 ) | 2.66 (1.16–6.13 ) | 0.48 (0.21–1.05 ) | 2.97 (2.31–3.83 ) | 2.64 (1.93–3.62 ) |
| No | 1.00 (0.91–1.10 ) | 1.10 (0.88–1.38 ) | 1.98 (1.62–2.42 ) | 1.28 (1.13–1.44 ) | 2.12 (1.99–2.26 ) | 2.33 (2.16–2.51 ) |
| **Coronary angiography** | | | | | | |
| Yes | 1.02 (0.85–1.21 ) | 1.29 (0.88–1.89 ) | 1.52 (1.07–2.16 ) | 0.96 (0.74–1.24 ) | 2.11 (1.91–2.34 ) | 2.32 (2.05–2.62 ) |
| No | 0.95 (0.86–1.05 ) | 0.95 (0.74–1.24 ) | 2.13 (1.74–2.61 ) | 1.14 (1.00–1.29 ) | 1.70 (1.59–1.81 ) | 1.89 (1.75–2.03 ) |
| **Percutaneus coronary intervention** | | | | | | |
| Yes | 1.14 (0.88–1.47 ) | 1.03 (0.55–1.91 ) | 1.31 (0.76–2.27 ) | 0.98 (0.68–1.42 ) | 2.07 (1.77–2.41 ) | 2.06 (1.70–2.49 ) |
| No | 0.90 (0.82–0.98 ) | 1.05 (0.84–1.32 ) | 2.06 (1.72–2.47 ) | 1.05 (0.93–1.18 ) | 1.68 (1.59–1.78 ) | 1.84 (1.72–1.97 ) |
| **Implantable cardioverter defibrillator** | | | | | | |
| Yes | 0.84 (0.63–1.11 ) | 1.22 (0.66–2.28 ) | 1.32 (0.74–2.35 ) | 0.76 (0.47–1.22 ) | 1.99 (1.66–2.39 ) | 2.47 (1.99–3.07 ) |
| No | 1.09 (0.95–1.26 ) | 1.32 (0.93–1.87 ) | 2.84 (2.15–3.75 ) | 1.29 (1.07–1.55 ) | 2.73 (2.48–3.00 ) | 2.59 (2.31–2.90 ) |
| **ICU admission** | | | | | | |
| Yes | 0.97 (0.81–1.17 ) | 1.50 (1.00–2.26 ) | 2.76 (2.00–3.79 ) | 1.18 (0.91–1.53 ) | 3.19 (2.85–3.57 ) | 2.73 (2.38–3.14 ) |
| No | 1.09 (0.91–1.29 ) | 1.13 (0.72–1.77 ) | 1.70 (1.13–2.56 ) | 1.19 (0.95–1.50 ) | 1.93 (1.70–2.19 ) | 2.42 (2.10–2.79 ) |
| **Ventilator treatment** | | | | | | |
| Yes | 0.98 (0.80–1.20 ) | 1.55 (1.00–2.39 ) | 2.45 (1.72–3.50 ) | 1.06 (0.79–1.41 ) | 3.22 (2.85–3.63 ) | 2.65 (2.28–3.08 ) |
| No | 1.07 (0.91–1.26 ) | 1.16 (0.76–1.75 ) | 2.08 (1.46–2.97 ) | 1.26 (1.02–1.56 ) | 2.05 (1.82–2.30 ) | 2.53 (2.22–2.89 ) |
| **Inotropics** | | | | | | |
| Yes | 0.96 (0.76–1.21 ) | 1.67 (1.02–2.74 ) | 3.11 (2.10–4.61 ) | 1.00 (0.72–1.39 ) | 3.28 (2.86–3.75 ) | 2.53 (2.13–3.01 ) |
| No | 1.07 (0.92–1.25 ) | 1.14 (0.78–1.67 ) | 1.89 (1.36–2.61 ) | 1.28 (1.05–1.57 ) | 2.17 (1.95–2.41 ) | 2.60 (2.30–2.94 ) |
| **Dialysis** | | | | | | |
| Yes | 1.47 (0.80–2.71 ) | 6.66(0.43–102.72) | 8.43 (1.62–43.81 ) | 1.12 (0.44–2.81 ) | 3.73 (2.60–5.34 ) | 2.44 (1.53–3.89 ) |
| No | 1.02 (0.89–1.16 ) | 1.22 (0.90–1.66 ) | 2.24 (1.74–2.88 ) | 1.19 (1.00–1.42 ) | 2.46 (2.26–2.68 ) | 2.57 (2.33–2.85 ) |

The original matching was maintained for shockable cardiac rhythm, therapeutic hypothermia, ICU admission, ventilator treatment, inotropics, and dialysis. For preexisting cardiac disease, coronary angiography, and percutaneous coronary intervention, the original matching was dissolved and an adjusted Cox regression analysis was performed for each stratum.
[a]Preexisting cardiac disease includes heart failure, coronary artery disease, atrial fibrillation or flutter, valvular heart disease, and peripheral vascular disease. ICD-8 and ICD-10 codes are provided in Supplementary Table 1.

© 2022 Secher N et al. *JAMA Network Open*.

**eTable 6.** Hazard ratios for neurological and psychiatric outcomes among patients with cardiac arrest compared with the general population, stratified by matching factors, socioeconomic status, length of stay, and comorbidity.

| | Cardiac arrest cohort *vs* matched general population cohort. Adjusted overall hazard ratio (95% CI) | | | | | |
|---|---|---|---|---|---|---|
| | Ischemic stroke Overall | Hemorrhagic stroke | Epilepsy | All-cause dementia | Mood disorders (incl. depression) | Anxiety |
| **Age, years** | | | | | | |
| <60 | 1.52 (1.18–1.95) | 2.89 (1.75–4.75 ) | 4.51 (3.17–6.42 ) | 3.12 (1.79–5.43) | 2.13 (1.91–2.38 ) | 2.51 (2.20–2.87) |
| 60-69 | 1.31 (1.06–1.61 ) | 0.89 (0.51–1.56 ) | 1.94 (1.26–3.00 ) | 1.65 (1.18–2.32 ) | 2.29 (2.00–2.62 ) | 2.49 (2.13–2.92) |
| 70-79 | 1.17 (0.98–1.39 ) | 1.18 (0.77–1.80 ) | 1.48 (0.97–2.25 ) | 1.29 (1.07–1.57 ) | 1.75 (1.55–1.98 ) | 1.82 (1.56–2.12 ) |
| ≥80 | 1.20 (0.99–1.44 ) | 1.11 (0.67–1.85 ) | 2.29 (1.38–3.81 ) | 1.10 (0.90–1.35 ) | 1.41 (1.22–1.62 ) | 1.51 (1.27–1.80 ) |
| **Sex** | | | | | | |
| Male | 1.52 (1.29–1.80 ) | 1.55 (1.03–2.34 ) | 2.77 (1.94–3.97 ) | 1.52 (1.26–1.83 ) | 1.90 (1.72–2.11 ) | 2.00 (1.77–2.26 ) |
| Female | 1.22 (1.08–1.37 ) | 1.25 (0.95–1.64 ) | 2.42 (1.92–3.05 ) | 1.31 (1.12–1.54 ) | 1.94 (1.80–2.10 ) | 2.28 (2.07–2.50 ) |
| **Calendar period** | | | | | | |
| 1996-2005 | 1.29 (1.13–1.47 ) | 1.15 (0.82–1.60 ) | 1.93 (1.41–2.64 ) | 1.42 (1.21–1.67 ) | 1.62 (1.49–1.77 ) | 1.99 (1.80–2.21 ) |
| 2006-2016 | 1.32 (1.14–1.52 ) | 1.58 (1.16–2.16 ) | 3.03 (2.36–3.90 ) | 1.34 (1.11–1.61 ) | 2.30 (2.11–2.50 ) | 2.29 (2.06–2.54 ) |
| **Income** | | | | | | |
| Low | 1.27 (1.10–1.47 ) | 1.01 (0.70–1.46 ) | 2.18 (1.60–2.97 ) | 1.21 (1.01–1.45 ) | 1.59 (1.44–1.75 ) | 1.84 (1.65–2.06 ) |
| Intermediate | 1.34 (1.15–1.57 ) | 1.17 (0.79–1.73 ) | 1.73 (1.25–2.39 ) | 1.25 (1.03–1.51 ) | 1.61 (1.45–1.78 ) | 1.70 (1.51–1.91 ) |
| High | 1.21 (0.97–1.50 ) | 1.65 (1.09–2.49 ) | 2.68 (1.85–3.88 ) | 1.23 (0.93–1.63 ) | 1.92 (1.71–2.15 ) | 1.99 (1.73–2.29 ) |
| Very high | 1.35 (1.04–1.74 ) | 1.51 (0.88–2.60 ) | 4.01 (2.64–6.08 ) | 1.97 (1.36–2.85 ) | 2.35 (2.05–2.70 ) | 2.68 (2.29–3.14 ) |
| **Length of stay, weeks** | | | | | | |
| <1 | 1.12 (0.94–1.34 ) | 0.96 (0.61–1.52 ) | 2.45 (1.65–3.62 ) | 1.46 (1.17–1.81 ) | 1.44 (1.28–1.62 ) | 1.99 (1.74–2.28 ) |
| 1-2 | 1.29 (1.07–1.57 ) | 1.61 (1.04–2.51 ) | 1.48 (0.93–2.38 ) | 0.95 (0.73–1.24 ) | 1.44 (1.27–1.64 ) | 1.89 (1.63–2.20 ) |
| 2-3 | 1.57 (1.24–1.98 ) | 1.47 (0.82–2.63 ) | 1.60 (0.97–2.66 ) | 1.16 (0.84–1.60 ) | 1.73 (1.48–2.02 ) | 2.02 (1.67–2.45 ) |
| 3-4 | 1.18 (0.83–1.68 ) | 1.40 (0.55–3.56 ) | 1.90 (0.93–3.88 ) | 1.16 (0.73–1.84 ) | 2.03 (1.64–2.51 ) | 1.75 (1.34–2.28 ) |
| ≥4 | 1.63 (1.31–2.03 ) | 1.83 (1.13–2.96 ) | 5.66 (3.83–8.36 ) | 2.34 (1.81–3.03 ) | 3.84 (3.38–4.35 ) | 3.07 (2.63–3.60 ) |
| **CCI score** | | | | | | |
| Normal | 1.33 (1.20–1.47 ) | 1.13 (0.88–1.44 ) | 2.43 (1.99–2.97 ) | 1.29 (1.13–1.47 ) | 1.72 (1.62–1.83 ) | 1.96 (1.82–2.11 ) |
| Moderate | 1.02 (0.76–1.37 ) | 1.81 (0.99–3.30 ) | 2.10 (1.25–3.52 ) | 1.65 (1.18–2.29 ) | 1.96 (1.65–2.33 ) | 1.94 (1.57–2.38 ) |
| Severe | 1.31 (0.99–1.74 ) | 1.43 (0.76–2.68 ) | 2.37 (1.36–4.14 ) | 0.86 (0.60–1.24 ) | 1.89 (1.58–2.25 ) | 1.95 (1.60–2.37 ) |
| Very severe | 1.06 (0.65–1.73 ) | 1.77 (0.60–5.21 ) | 2.87 (1.15–7.17 ) | 1.94 (1.11–3.39 ) | 1.79 (1.32–2.44 ) | 1.54 (1.10–2.14 ) |
| **Education** | | | | | | |
| Basic education, primary school | 1.32 (1.09–1.59 ) | 1.39 (0.91–2.11 ) | 2.62 (1.86–3.70 ) | 1.21 (0.95–1.53 ) | 1.81 (1.61–2.02 ) | 1.94 (1.70–2.22 ) |
| Youth education, high school, or similar education | 1.23 (1.00–1.51 ) | 1.27 (0.81–1.98 ) | 2.74 (1.93–3.88 ) | 1.29 (0.97–1.70 ) | 2.20 (1.96–2.48 ) | 2.18 (1.89–2.51 ) |
| Higher education | 1.24 (0.87–1.75 ) | 1.36 (0.60–3.07 ) | 3.87 (2.31–6.49 ) | 1.18 (0.75–1.87 ) | 1.91 (1.58–2.32 ) | 1.63 (1.30–2.03 ) |
| Missing | 1.91 (1.25–2.91 ) | 2.32 (0.76–7.01 ) | 4.80 (1.52–15.11 ) | 1.81 (1.09–3.01 ) | 1.94 (1.41–2.65 ) | 2.23 (1.59–3.12 ) |

The original matching was maintained for age, sex, calendar period, and length of stay. For income, CCI score, and education, the original matching was dissolved and an adjusted Cox regression analysis was performed for each stratum. CCI indicates Charlson Comorbidity Index.

© 2022 Secher N et al. *JAMA Network Open*.

**eTable 7.** Hazard ratios for neurological and psychiatric outcomes among patients with cardiac arrest compared with the general population, stratified by comorbidity and treatment interventions.

| | Cardiac arrest cohort *vs* matched general population cohort. Adjusted overall hazard ratio (95% CI) | | | | | |
|---|---|---|---|---|---|---|
| | Ischemic stroke | Hemorrhagic stroke | Epilepsy | All-cause dementia | Mood disorders (incl. depression) | Anxiety |
| **Shockable cardiac rhythm** | | | | | | |
| Yes | 1.21 (1.05–1.41 ) | 1.12 (0.77–1.62 ) | 1.72 (1.19–2.47 ) | 1.20 (0.99–1.44 ) | 1.64 (1.49–1.80 ) | 1.87 (1.67–2.11 ) |
| **Preexsisting cardiac disease[a]** | | | | | | |
| Yes | 1.17 (1.05–1.30 ) | 1.02 (0.78–1.32 ) | 1.59 (1.24–2.04 ) | 1.13 (0.98–1.30 ) | 1.44 (1.34–1.55 ) | 1.71 (1.57–1.87 ) |
| No | 1.45 (1.25–1.68 ) | 1.51 (1.09–2.09 ) | 3.17 (2.54–3.95 ) | 1.64 (1.38–1.95 ) | 2.18 (2.02–2.35 ) | 2.24 (2.05–2.45 ) |
| **Preexisting chronic pulmonary disease** | | | | | | |
| Yes | 1.43 (1.16–1.76 ) | 1.09 (0.66–1.81 ) | 3.58 (2.48–5.16 ) | 1.12 (0.85–1.48 ) | 1.76 (1.56–1.99 ) | 1.77 (1.54–2.04 ) |
| No | 1.26 (1.08–1.47 ) | 1.51 (1.09–2.10 ) | 2.54 (1.94–3.34 ) | 1.38 (1.13–1.67 ) | 2.09 (1.91–2.29 ) | 2.15 (1.93–2.39 ) |
| **Therapeutic hypothermia** | | | | | | |
| Yes | 1.82 (1.17–2.85 ) | 1.96 (0.71–5.39 ) | 3.23 (1.49–6.97 ) | 0.59 (0.24–1.46 ) | 2.78 (2.17–3.55 ) | 3.05 (2.25–4.12 ) |
| No | 1.29 (1.17–1.43 ) | 1.31 (1.04–1.66 ) | 2.48 (2.03–3.04 ) | 1.40 (1.24–1.59 ) | 1.88 (1.77–2.00 ) | 2.10 (1.94–2.26 ) |
| **Coronary angiography** | | | | | | |
| Yes | 1.15 (0.96–1.38 ) | 1.73 (1.19–2.53 ) | 1.92 (1.36–2.72 ) | 1.07 (0.84–1.37 ) | 2.00 (1.80–2.22 ) | 2.03 (1.78–2.32 ) |
| No | 1.39 (1.24–1.56 ) | 1.17 (0.87–1.56 ) | 2.85 (2.25–3.61 ) | 1.52 (1.32–1.75 ) | 1.89 (1.75–2.04 ) | 2.19 (2.01–2.40 ) |
| **Percutaneus coronory intervention** | | | | | | |
| Yes | 1.21 (0.94–1.57 ) | 2.28 (1.29–4.03 ) | 1.87 (1.10–3.18 ) | 1.40 (1.01–1.94 ) | 2.21 (1.91–2.56 ) | 1.87 (1.54–2.27 ) |
| No | 1.33 (1.20–1.48 ) | 1.26 (0.98–1.62 ) | 2.74 (2.22–3.39 ) | 1.40 (1.22–1.59 ) | 1.89 (1.76–2.02 ) | 2.21 (2.04–2.39 ) |
| **Implantable cardioverter defibrillator** | | | | | | |
| Yes | 1.11 (0.80–1.53 ) | 1.87 (0.95–3.68 ) | 2.42 (1.31–4.47 ) | 0.90 (0.52–1.53 ) | 1.41 (1.16–1.72 ) | 1.80 (1.41–2.29 ) |
| No | 1.39 (1.19–1.61 ) | 1.43 (1.02–2.00 ) | 3.25 (2.50–4.24 ) | 1.42 (1.19–1.71 ) | 2.48 (2.26–2.71 ) | 2.34 (2.10–2.62 ) |
| **ICU admission** | | | | | | |
| Yes | 1.42 (1.17–1.72 ) | 1.79 (1.20–2.68 ) | 4.10 (3.00–5.60 ) | 1.37 (1.06–1.78 ) | 2.74 (2.46–3.06 ) | 2.64 (2.30–3.02 ) |
| No | 1.23 (1.02–1.48 ) | 1.20 (0.75–1.91 ) | 1.92 (1.27–2.89 ) | 1.25 (1.00–1.58 ) | 1.65 (1.45–1.87 ) | 1.82 (1.57–2.11 ) |
| **Ventilator treatment** | | | | | | |
| Yes | 1.41 (1.14–1.75 ) | 2.18 (1.40–3.39 ) | 4.27 (3.06–5.96 ) | 1.23 (0.92–1.64 ) | 2.77 (2.47–3.11 ) | 2.58 (2.23–2.99 ) |
| No | 1.26 (1.06–1.50 ) | 1.12 (0.73–1.72 ) | 2.17 (1.50–3.14 ) | 1.33 (1.08–1.65 ) | 1.76 (1.57–1.97 ) | 1.95 (1.70–2.24 ) |
| **Inotropics** | | | | | | |
| Yes | 1.32 (1.03–1.69 ) | 1.88 (1.14–3.10 ) | 4.28 (2.97–6.17 ) | 1.17 (0.84–1.64 ) | 2.85 (2.49–3.25 ) | 2.66 (2.25–3.14 ) |
| No | 1.32 (1.12–1.54 ) | 1.34 (0.92–1.97 ) | 2.39 (1.71–3.33 ) | 1.36 (1.12–1.66 ) | 1.87 (1.69–2.08 ) | 2.01 (1.77–2.28 ) |
| **Dialysis** | | | | | | |
| Yes | 1.38 (0.70–2.72 ) | 2.10 (0.45–9.83 ) | 2.83 (0.54–14.86 ) | 1.93 (0.70–5.30 ) | 3.97 (2.69–5.86 ) | 2.60 (1.59–4.26 ) |
| No | 1.31 (1.15–1.51 ) | 1.49 (1.10–2.02 ) | 2.84 (2.22–3.64 ) | 1.29 (1.09–1.54 ) | 2.12 (1.95–2.30 ) | 2.21 (2.00–2.45 ) |

The original matching was maintained for all strata except preexisting cardiac disease, as treatment codes were only accessed for the cardiac arrest cohort. For preexisting cardiac disease, the original matching was dissolved and an adjusted Cox regression analysis was performed for each stratum.

[a]Preexisting cardiac disease included heart failure, coronary artery disease, atrial fibrillation or flutter, valvular heart disease, and peripheral vascular disease. ICD-8 and ICD-10 codes are provided in Supplementary Table 1.

© 2022 Secher N et al. *JAMA Network Open.*

**eTable 8.** Sensitivity analyses examining risk of stroke outcomes among patients with cardiac arrest and people in the matched comparison cohorts. Computed tomography or magnetic resonance imaging were performed during the hospital admission.

| | Cardiac arrest cohort | | Matched MI cohort | | Matched general population cohort | | Cardiac arrest cohort *vs* matched MI cohort | Cardiac arrest cohort *vs* matched general population cohort |
|---|---|---|---|---|---|---|---|---|
| Time since disharge | Outcomes/ No. at risk | Cumulative incidence per 1000 persons (95% CI)[a] | Outcomes/ No. at risk | Cumulative incidence per 1000 persons (95% CI)[a] | Outcomes/ No. at risk | Cumulative incidence per 1000 persons (95% CI)[a] | Adjusted hazard ratio (95% CI)[b] | Adjusted hazard ratio (95% CI)[b] |
| **Ischemic stroke** | | | | | | | | |
| 0-3 months | 26/10,127 | 2.60 (1.7–3.8 ) | 150/104,305 | 1.46 (1.2–1.7 ) | 91/106,651 | 0.86 (0.7–1.1 ) | 1.44 (0.86–2.40 ) | 2.86 (1.66–4.93 ) |
| 4-12 months | 43/9037 | 4.91 (3.6–6.6 ) | 417/100,598 | 4.29 (3.9–4.7 ) | 314/103,482 | 3.14 (2.8–3.5 ) | 1.26 (0.89–1.79 ) | 1.48 (1.02–2.14 ) |
| 13-60 months | 121/7775 | 18.73 (15.6–22.3 ) | 1621/90,086 | 21.95 (20.9–23.0 ) | 1291/94,026 | 16.75 (15.9–17.7 ) | 0.85 (0.69–1.04 ) | 1.04 (0.84–1.29 ) |
| >5 years | 94/3699 | 51.26 (39.3–65.5 ) | 1285/46,320 | 62.37 (57.4–67.6 ) | 1093/51,974 | 45.91 (42.2–49.8 ) | 0.91 (0.71–1.17 ) | 1.23 (0.96–1.59 ) |
| Overall | 284/10,127 | 54.76 (46.5–64.0 ) | 3473/104,305 | 74.45 (70.5–78.6 ) | 2789/106,651 | 58.43 (55.2–61.8 ) | 0.97 (0.84–1.11 ) | 1.24 (1.08–1.44 ) |
| **Hemorrhagic stroke** | | | | | | | | |
| 0-3 months | 12/10,586 | 1.14 (0.6–2.0 ) | 21/105,387 | 0.20 (0.1–0.3 ) | 34/107,236 | 0.32 (0.2–0.4 ) | 9.30 (2.62–33.04 ) | 6.08 (2.32–15.94 ) |
| 4-12 months | 14/9445 | 1.53 (0.9–2.5 ) | 70/101,715 | 0.71 (0.6–0.9 ) | 67/104,091 | 0.66 (0.5–0.8 ) | 3.06 (1.44–6.47 ) | 2.47 (1.24–4.91 ) |
| 13-60 months | 25/8119 | 3.69 (2.5–5.4 ) | 269/91,300 | 3.57 (3.2–4.0 ) | 291/94,761 | 3.73 (3.3–4.2 ) | 0.85 (0.54–1.36 ) | 0.96 (0.59–1.56 ) |
| >5 years | 29/3878 | 14.85 (9.4–22.4 ) | 247/47,543 | 11.70 (9.9–13.8 ) | 254/52,819 | 10.40 (8.8–12.3 ) | 1.28 (0.79–2.07 ) | 1.87 (1.14–3.06 ) |
| Overall | 80/10,586 | 14.91 (11.1–19.7 ) | 607/105,387 | 13.44 (11.9–15.1 ) | 646/107,236 | 13.36 (11.9–15.0 ) | 1.33 (1.01–1.75 ) | 1.58 (1.20–2.09 ) |

[a]Per 1000 persons.
[b]Controlled for age, sex, calendar year, and adjusted for the variables in Table 1.

© 2022 Secher N et al. *JAMA Network Open*.

**eTable 9.** Sensitivity analyses examining risk of specified and unspecified ischemic stroke among patients with cardiac arrest and people in the matched comparison cohorts.

| | Cardiac arrest cohort | | Matched MI cohort | | Matched general population cohort | | Cardiac arrest cohort *vs* matched MI cohort | Cardiac arrest cohort *vs* matched general population cohort |
|---|---|---|---|---|---|---|---|---|
| Time since disharge | Outcomes/ No. at risk | Cumulative incidence per 1000 persons (95% CI)[a] | Outcomes/ No. at risk | Cumulative incidence per 1000 persons (95% CI)[a] | Outcomes/ No. at risk | Cumulative incidence per 1000 persons (95% CI)[a] | Adjusted hazard ratio (95% CI)[b] | Adjusted hazard ratio (95% CI)[b] |
| **Specified ischemic stroke** | | | | | | | | |
| 0-3 months | 23/11,366 | 2.04 (1.3–3.0 ) | 163/117,003 | 1.41 (1.2–1.6 ) | 104/119,703 | 0.88 (0.7–1.1 ) | 1.20 (0.72–2.01 ) | 2.39 (1.37–4.17 ) |
| 4-12 months | 43/10,181 | 4.33 (3.2–5.8 ) | 472/113,082 | 4.30 (3.9–4.7 ) | 369/116,405 | 3.27 (3.0–3.6 ) | 1.08 (0.77–1.52 ) | 1.15 (0.80–1.64 ) |
| 13-60 months | 135/8792 | 17.85 (15.0–21.0 ) | 1983/101,865 | 23.15 (22.1–24.2 ) | 1496/106,538 | 16.69 (15.9–17.5 ) | 0.75 (0.62–0.91 ) | 1.03 (0.84–1.26 ) |
| >5 years | 133/4384 | 58.40 (47.1–71.3 ) | 2252/55,157 | 106.66 (89.3–125.8 ) | 1852/62,431 | 67.45 (62.8–72.3 ) | 0.77 (0.63–0.94 ) | 1.11 (0.90–1.36 ) |
| Overall | 334/11,366 | 56.75 (49.2–65.0 ) | 4870/117,003 | 108.46 (95.1–122.8 ) | 3821/119,703 | 76.15 (72.2–80.2 ) | 0.82 (0.72–0.93 ) | 1.12 (0.98–1.28 ) |
| **Unspecified ischemic stroke** | | | | | | | | |
| 0-3 months | 38/11,366 | 3.37 (2.4–4.6 ) | 135/117,003 | 1.17 (1.0–1.4 ) | 63/119,703 | 0.53 (0.4–0.7 ) | 2.61 (1.69–4.02 ) | 5.48 (3.13–9.58 ) |
| 4-12 months | 53/10,173 | 5.35 (4.1–6.9 ) | 353/113,106 | 3.22 (2.9–3.6 ) | 235/116,449 | 2.08 (1.8–2.4 ) | 1.55 (1.11–2.16 ) | 2.05 (1.41–2.98 ) |
| 13-60 months | 127/8781 | 17.15 (14.4–20.3 ) | 1408/102,028 | 16.42 (15.6–17.3 ) | 1035/106,711 | 11.53 (10.8–12.2 ) | 1.04 (0.85–1.28 ) | 1.16 (0.93–1.45 ) |
| >5 years | 103/4384 | 48.33 (35.9–63.3 ) | 1203/55,540 | 43.48 (40.1–47.1 ) | 1038/62,712 | 32.91 (30.3–35.7 ) | 1.10 (0.86–1.41 ) | 1.53 (1.18–1.98 ) |
| Overall | 321/11,366 | 52.31 (43.9–61.7 ) | 3099/117,003 | 53.23 (50.5–56.1 ) | 2371/119,703 | 41.23 (38.9–43.7 ) | 1.23 (1.08–1.41 ) | 1.54 (1.33–1.77 ) |

[a]Per 1000 persons.
[b]Controlled for age, sex, calendar year, and adjusted for the variables in Table 1.

© 2022 Secher N et al. *JAMA Network Open*.

**eTable 10.** Sensitivity analyses examining risk of first-time diagnosis of mood disorders among patients with cardiac arrest and people in the matched comparison cohorts.

| | Cardiac arrest cohort | | Matched MI cohort | | Matched general population cohort | | Cardiac arrest cohort *vs* matched MI cohort | Cardiac arrest cohort *vs* matched general population cohort |
|---|---|---|---|---|---|---|---|---|
| Time since disharge | Outcomes/ No. at risk | Cumulative incidence per 1000 persons (95% CI)[a] | Outcomes/ No. at risk | Cumulative incidence per 1000 persons (95% CI)[a] | Outcomes/ No. at risk | Cumulative incidence per 1000 persons (95% CI)[a] | Adjusted hazard ratio (95% CI)[b] | Adjusted hazard ratio (95% CI)[b] |
| **First-time diagnosis of mood disorders including depression** | | | | | | | | |
| 0-3 months | 29/10,412 | 2.85 (2.0–4.1 ) | 45/103,764 | 0.44 (0.3–0.6 ) | 28/109,953 | 0.26 (0.2–0.4 ) | 6.94 (3.66–13.15 ) | 23.94 (9.25–61.97 ) |
| 4-12 months | 24/9060 | 2.79 (1.8–4.1 ) | 107/100,025 | 1.12 (0.9–1.3 ) | 81/106,721 | 0.79 (0.6–1.0 ) | 2.73 (1.52–4.91 ) | 3.32 (1.76–6.28 ) |
| 13-60 months | 24/7428 | 3.92 (2.6–5.8 ) | 341/88,750 | 4.75 (4.3–5.3 ) | 315/96,628 | 4.01 (3.6–4.5 ) | 0.77 (0.47–1.25 ) | 1.10 (0.68–1.79 ) |
| >5 years | 18/3421 | 11.48 (6.5–19.1 ) | 322/45,810 | 15.70 (13.4–18.2 ) | 239/54,291 | 10.39 (8.8–12.2 ) | 0.87 (0.50–1.54 ) | 1.32 (0.72–2.43 ) |
| Overall | 95/10,412 | 16.31 (12.2–21.3 ) | 815/103,764 | 18.75 (16.8–20.8 ) | 663/109,953 | 13.98 (12.5–15.6 ) | 1.54 (1.19–2.00 ) | 2.18 (1.67–2.85 ) |

[a]Per 1000 persons.
[b]Controlled for age, sex, calendar year, and adjusted for the variables in Table 1.

© 2022 Secher N et al. *JAMA Network Open*.

**eTable 11.** Sensitivity analysis examining the risk of depression (excluding other mood disorders) among patients with cardiac arrest and people in the matched comparison cohorts

| | Cardiac arrest cohort | | Matched MI cohort | | Matched general population cohort | | Cardiac arrest cohort *vs* matched MI cohort | Cardiac arrest cohort *vs* matched general population cohort |
|---|---|---|---|---|---|---|---|---|
| Time since disharge | Outcomes/ No. at risk | Cumulative incidence per 1000 persons (95% CI)[a] | Outcomes/ No. at risk | Cumulative incidence per 1000 persons (95% CI)[a] | Outcomes/ No. at risk | Cumulative incidence per 1000 persons (95% CI)[a] | Adjusted hazard ratio (95% CI) [b] | Adjusted hazard ratio (95% CI) [b] |
| **Depression** | | | | | | | | |
| 0-3 months | 61/11,748 | 5.25 (4.1–6.7) | 133/117,488 | 1.15 (1.0–1.4) | 79/119,862 | 0.67 (0.5–0.8) | 5.82 (3.86–8.77) | 8.99 (5.55–14.56) |
| 4-12 months | 71/10,479 | 6.94 (5.5–8.7) | 378/113,512 | 3.44 (3.1–3.8) | 275/116,580 | 2.43 (2.2–2.7) | 2.20 (1.61–3.01) | 2.33 (1.69–3.22) |
| 13-60 months | 154/9015 | 20.09 (17.1–23.4) | 1548/102,272 | 18.02 (17.1–18.9) | 1293/106,756 | 14.55 (13.8–15.4) | 1.09 (0.90–1.33) | 1.36 (1.12–1.66) |
| >5 years | 108/4447 | 45.68 (36.1–56.9) | 1626/55,508 | 61.59 (57.3–66.0) | 1592/62,548 | 54.18 (50.9–57.6) | 0.90 (0.71–1.14) | 0.94 (0.74–1.19) |
| Overall | 394/11,748 | 55.81 (49.1–63.1) | 3685/117,488 | 68.45 (65.1–71.9) | 3239/119,862 | 62.13 (59.3–65.1) | 1.34 (1.18–1.52) | 1.51 (1.33–1.71) |

[a]Per 1000 persons

[b]Controlled for age, sex, calendar year, and adjusted for the variables in Table 1.

© 2022 Secher N et al. *JAMA Network Open*.

**eTable 12.** Overall risk of neurological and psychiatric outcomes among cardiac arrest patients and ICU patients in the matched comparison cohort.

| | Cardiac arrest cohort | | Matched ICU cohort | | Cardiac arrest cohort *vs* matched ICU cohort |
|---|---|---|---|---|---|
| | Outcomes/ No. at risk | Cumulative incidence per 1000 persons (95% CI)[a] | Outcomes/ No. at risk | Cumulative incidence per 1000 persons (95% CI)[a] | Adjusted hazard ratio (95% CI)[b] |
| **Ischemic stroke** | 434/9383 | 91.96 (78.3–106.9) | 4116/89,864 | 131.94 (114.6–150.5) | 0.91 (0.79–1.04) |
| **Hemorrhagic stroke** | 94/9784 | 17.79 (13.8–22.6) | 1021/90,861 | 24.49 (21.1–28.3) | 0.79 (0.59–1.05) |
| **Epilepsy** | 171/9450 | 30.90 (25.6–36.9) | 1212/86,476 | 44.93 (32.7–59.9) | 1.83 (1.46–2.29) |
| **Dementia** | 259/8122 | 64.42 (54.9–75.0) | 2408/81,738 | 67.18 (63.5–71.0) | 1.42 (1.18–1.72) |
| **Parkinson's disease** | 28/8314 | 10.66 (6.5–16.6) | 318/83,415 | 10.19 (8.6–12.0) | 0.70 (0.40–1.24) |
| **Mood disorders including depression** | 1411/8492 | 239.41 (223.8–255.3) | 9000/73,850 | 209.37 (202.7–216.2) | 1.50 (1.39–1.62) |
| **Anxiety** | 1019/9052 | 164.72 (151.3–178.6) | 6084/84,200 | 121.22 (117.4–125.1) | 1.72 (1.57–1.89) |

[a]Per 1000 persons
[b]Controlled for age, sex, calendar year, and adjusted for the variables in Table 1.

© 2022 Secher N et al. *JAMA Network Open*.
